# Confinement Drives Valley Splitting above 4 K in Buried Silicon Quantum Wells

**DOI:** 10.1021/acs.nanolett.6c04172

**Published:** 2026-10-22

**Authors:** Davide Degli Esposti, Emma C. Brann, Asser Elsayed, Davide Costa, Mark Friesen, Giordano Scappucci

**Affiliations:** † QuTech and Kavli Institute of Nanoscience, 2860 Delft University of Technology, Lorentzweg 1, 2628 CJ Delft, The Netherlands; ‡ Department of Physics, 5228 University of Wisconsin-Madison, Madison, Wisconsin 53706, United States

**Keywords:** Si/SiGe, quantum wells, valley splitting, orbital energy, quantum dots, spin qubits

## Abstract

Controlling the energy scales of a quantum system is essential for defining robust qubits. In silicon, the nearly degenerate conduction-band valleys create a leakage channel from the single-spin computational basis. Here, we perform spectroscopic measurements of single- and two-electron states in buried silicon quantum wells. Across a linear array of four quantum dots with an average orbital energy of 2.4(2) meV, we report an average single-electron valley splitting of 0.40(6) meV and an average two-electron singlet–triplet splitting of 0.24­(7) meV. In three dots, we observe a strong correlation between valley splitting and orbital energy, with an average linear coefficient of ≈0.22 (meV/meV). In contrast, the remaining dot exhibits the highest valley splitting of 0.76(2) meV and low correlation. Our findings show that strong confinement can effectively enhance valley splitting in buried quantum wells, thereby establishing a viable path toward shuttling and sparse-occupation-based architectures in low-disorder heterostructures.

## Graphical abstract

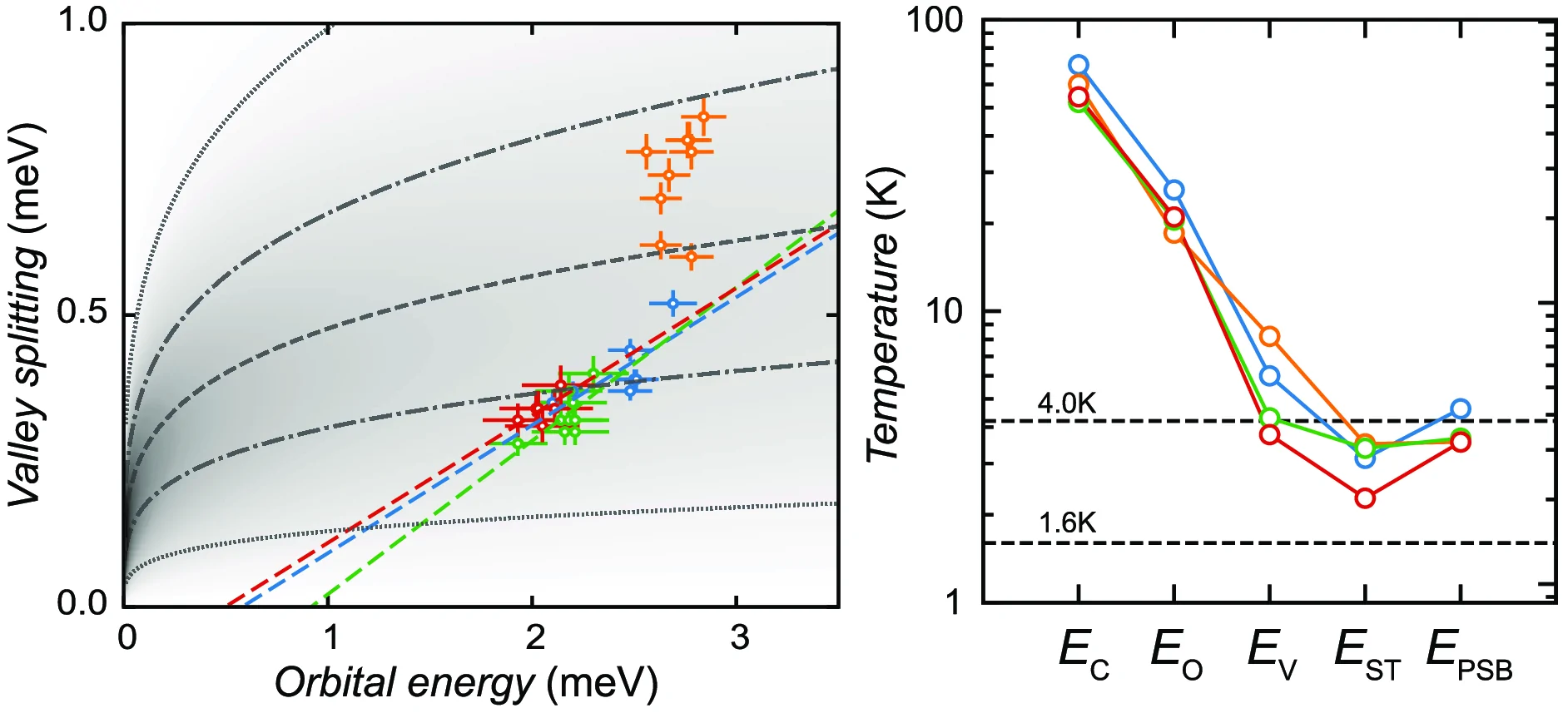

Gate-defined quantum dots in ^28^Si/SiGe heterostructures are a promising platform for building large-scale quantum processors.^1,2^ Indeed, the key building blocks of a large quantum processor, such as high-fidelity single and two-qubit operation,^3,4^ coherent shuttling and teleportation to distribute quantum information,^5–8^ prolonged digital quantum circuit operation,^9,10^ and first error correction schemes,^11^ have been demonstrated. Moreover, the advanced semiconductor industry proved to be able to manufacture uniform and reliable spin qubit devices.^12–19^ The buried isotopically purified quantum well ensures a quiet environment with low electrical and magnetic noise.^17,20^ Low disorder and the absence of decoherence hotspots will be a desirable requirement for scaling to large arrays and developing shuttling-based or sparse-array architectures.^11,21–27^ In this context, the uncontrollable and frequent occurrence of degenerate valley states near the computational single-spin basis remains a major obstacle for scaling to large arrays, connecting distant registers via shuttling links, and operating at high temperature.^28–38^

In the silicon metal-oxide-semiconductor (SiMOS), the strong electrostatic confinement at the semiconductor–dielectric interface has enabled gate-tunable valley splitting up to ≈0.8 meV^39^ and high-fidelity qubit operation at high temperature.^40,41^ On the contrary, Si/SiGe heterostructures are characterized by a shallower confinement due to the larger distance between the gates and the buried quantum well set by the epitaxial SiGe barrier. Multiple experimental and theoretical works investigating the microscopic origin of the valley separation in Si/SiGe demonstrate that alloy disorder, intended as the random distribution of Ge atoms in the SiGe alloy, constitutes the dominant factor limiting the valley splitting in these heterostructures below 100 μeV.^42–47^ As a result, different strategies have been proposed and are experimentally investigated to engineer the disorder potential and systematically enhance the valley splitting,^43^ including the use of unconventional heterostructures that incorporate Ge into the interior^35,42,48–51^ or the boundary of the quantum well.^52–54^ However, all of these strategies aim to increase alloy scattering within or near the quantum well, thereby compromising the low-disorder environment characteristic of these heterostructures, and have so far resulted in only nondeterministic improvements.^14^ Recently, low disorder and high valley splitting have been demonstrated in conventional ^28^Si/SiGe heterostructures.^30^ Such heterostructures enabled more complex devices to demonstrate high-fidelity spin shuttling^5^ and weight-four parity stabilizers,^11^ suggesting a possible cointegration of these characteristics.

Here, we advance our investigations of the same low-disorder shallow quantum wells and report spectroscopic measurements of single- and two-electron states in tightly confined quantum-dot devices embedded in these heterostructures. We measure all of the relevant energy scales for defining single-electron spin qubits, such as the charging energy (*E*_*C*_), the orbital energy (*E*_*O*_), the single-electron valley splitting (*E*_*V*_), and the two-electron singlet–triplet splitting via magnetospectroscopy (*E*_*ST*_) and Pauli-spin-blockade spectroscopy (*E*_*PSB*_). By using gates to tune the confinement, we find a strong correlation between *E*_*O*_ and *E*_*V*_ over multiple quantum dots, boosting *E*_*V*_ above 0.5 meV. This correlation breaks down in one quantum dot, which features both the largest *E*_*O*_ of 2.77(4) meV and *E*_*V*_ valley splitting of 0.76(2) meV. We compare our measurement with current theories of valley splitting in the alloy-disorder-dominated regime and reveal that confinement might be surprisingly efficient in these heterostructures at enhancing the single-electron valley splitting. Despite the tight confinement, we find a substantial reduction of the two-electron singlet–triplet splitting in the same dots compared to the respective valley splitting. This study underscores the importance of characterizing energy scales beyond single-electron states and provides a viable path to codesigning quantum dots and buried quantum wells with low disorder and consistently large valley splitting.

We fabricated quantum dot spin qubit devices on top of ^28^Si/SiGe heterostructures grown by reduced-pressure chemical vapor deposition (RP-CVD) on 100 mm Si(001) substrates^30,55^ using lift-off processes and overlapping gates^29^ (see Supporting Information, Methods). The growth protocol produces low-disorder quantum wells with mobility exceeding 3 × 10^5^ cm^2^/(V s)^30^ and excludes incorporation or diffusion of Ge within the 28Si well (Supporting Information, Methods), based on prior characterization of similar heterostructures by secondary ions mass spectrosopy and atom probe tomography in ref 42 and further electron-energy-loss-spectroscopy (EELS) in Supplementary Figure S2. We use shallow quantum wells buried 30 nm beneath the surface and thin high-k dielectrics in the gate stack to maximize the capacitive coupling between the charges in the quantum well and the topmost electrodes. From the measurement of the two-dimensional electron gas (2DEG), we extract an effective capacitance of *C*_2*D*EG_ ≃ 210 nF/cm^2^ ^55^ at the first gate layer. Figure 1(a) shows the device layout and the voltage configuration used to achieve the single-electron occupancy in the quantum dot array. The array comprises four quantum dots (P1–P4), a single charge sensor on the left side (S1), and a double charge sensor on the right side (S2). We design these devices with a tight gate pitch of 90 nm, a nominal single-dot plunger width of 40 nm, and strong in-plane asymmetry to lift the in-plane orbital states. We operate the right sensor, S2, as a single-charge sensor defined by the bottom plunger. We connect the two sensing dots to two off-chip high-inductance superconducting resonators to perform fast readout via frequency-multiplexed RF-reflectometry (see Supporting Information) and perform all experiments at a constant mixing chamber temperature of 150 mK to minimize drift and heating effects.^56^

**Figure 1:**
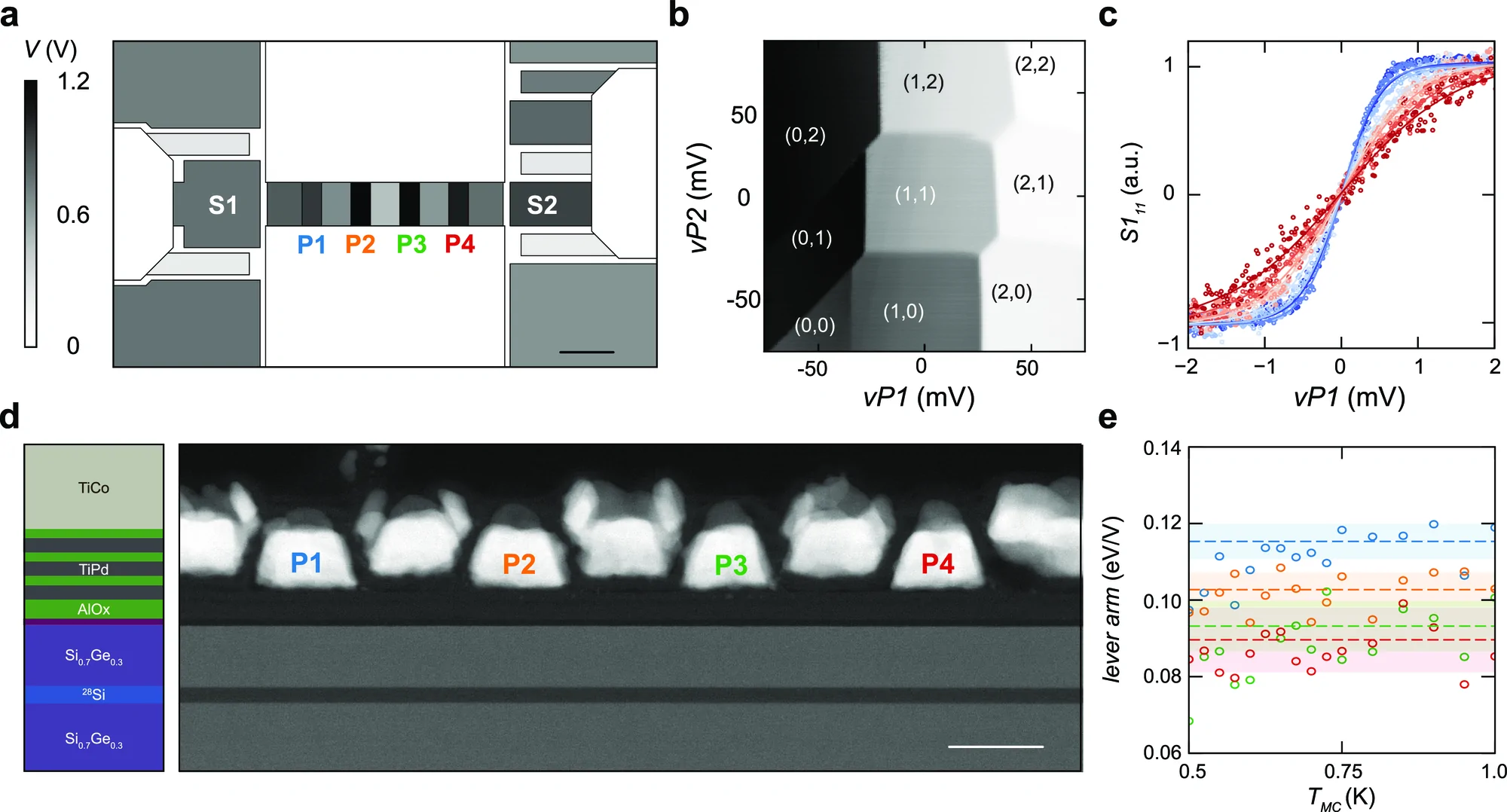
Heterostructure and quantum dot gate stack. (a) Quantum dot gate layout and voltage configuration used to achieve single-electron occupancy in the four-dot array. The top-right sensor is fully accumulated and used as an extension of the accumulation gate. The two sensors (S1 and S2) are used to sense the charge state of the four quantum dots (P1 to P4). The black line corresponds to 100 nm. (b) Double-dot charge stability diagram centered in the DC voltage configuration of (a) for the virtualized plunger gates *vP*_1_ and *vP*_2_. (c) First electron polarization line for virtual plunger vP1 measured as a function of mixing chamber temperature for temperatures between 10 mK (dark blue) and 1 K (dark red). The solid line is a fit to the theoretical model used to extract the lever arm. (d) Atomic-resolution high-angle annular dark-field (HAADF) (Z-contrast) scanning transmission electron microscopy (STEM) image of the overlapping gate and heterostructure stack taken along the linear quantum dot array. The gates corresponding to the quantum dot plungers are labeled P1–P4. The sketch on the left side highlights the layer order forming the heterostructure and gate stack. The white line corresponds to 50 nm. (e) Average lever arm (dashed line) and standard deviation (shaded area) for the four virtual plungers vP1 (blue), vP2 (orange), vP3 (green), and vP4 (red) obtained from the fitting of the first electron polarization line to the Fermi distribution for temperatures of the mixing chamber between 0.5 and 1 K.

We populate the array in the (1,1,1,1) electron configuration and estimate the cross-capacitance coupling between gates from the virtual-gate matrix. In the Supporting Information, we report the virtualization matrix for the main device and for a lithographically identical device to validate the reproducibility of the gate layout and cross-capacitive coupling. Figure 1(b) shows a charge stability diagram centered in the single-electron configuration for the virtualized plungers vP1 and vP2. The symmetry of the devices is confirmed by the high-resolution scanning transmission electron micrograph (STEM) image in Figure 1(d) of the plungers and barriers along the array direction. From the STEM image, we measure a plunger (barrier) width of 40 nm (50 nm) and confirm it by scanning electron microscopy and atomic force microscopy of lithographically identical devices (see Supporting Information).

We continue to characterize the quantum dot device and measure the lever arm of each virtualized plunger gate via temperature-activation broadening of the first electron addition line.^5^ We maintain a low tunnel coupling between the dots and measure the first-electron-addition line at temperatures well above this energy scale to ensure that the broadening is temperature limited. Figure 1(c) shows the first polarization line as a function of vP1 for temperatures of the mixing chamber between 10 mK and 1 K. We fit the addition line with the phenomenological relation *A* + *B* *V* + *C* tanh α­(*V* – *V*_0_) β where *A*, *B*, and α are fitting parameters and $\beta =\frac{e}{2k_{B}T_{e}}$ with *k*_*B*_ the Boltzmann constant, and *T*_*e*_ is the electron temperature that we approximate with the temperature of the mixing chamber in this regime. Figure 1(e) shows the results of the fit at temperatures between 0.5 and 1.0 K, where the addition line is mostly temperature broadened. The extracted lever arms remain constant in this regime and converge to values between 0.08 and 0.12 eV/V.

We start the characterization of the valley and orbital states by performing detuning axis pulsed spectroscopy (DAPS)^36^ on the entire array. We prepare one electron in one of the dots and track the resonant tunneling to the nearby dot across the (1,0) to (0,1) transition as a function of the relative detuning (*ve*_12_ and *ve*_43_) and use the previously measured lever arm to convert it into an energy spectrum. This technique allows us to extract the energy spectrum in a configuration in which the quantum dot array is strongly decoupled from the sensing dot, and the number of electrons in the array is constant. Figure 2(a) shows the results of this measurement for a single electron tunneling from P1 to P2 as a function of the waiting time (*t*_*wait*_) and detuning (*ve*_12_). We recognize two pairs of resonant tunneling states that we attribute to the first valleys and orbital states^36^ and use the separations to extract *E*_*V*_ and *E*_*O*_.

**Figure 2:**
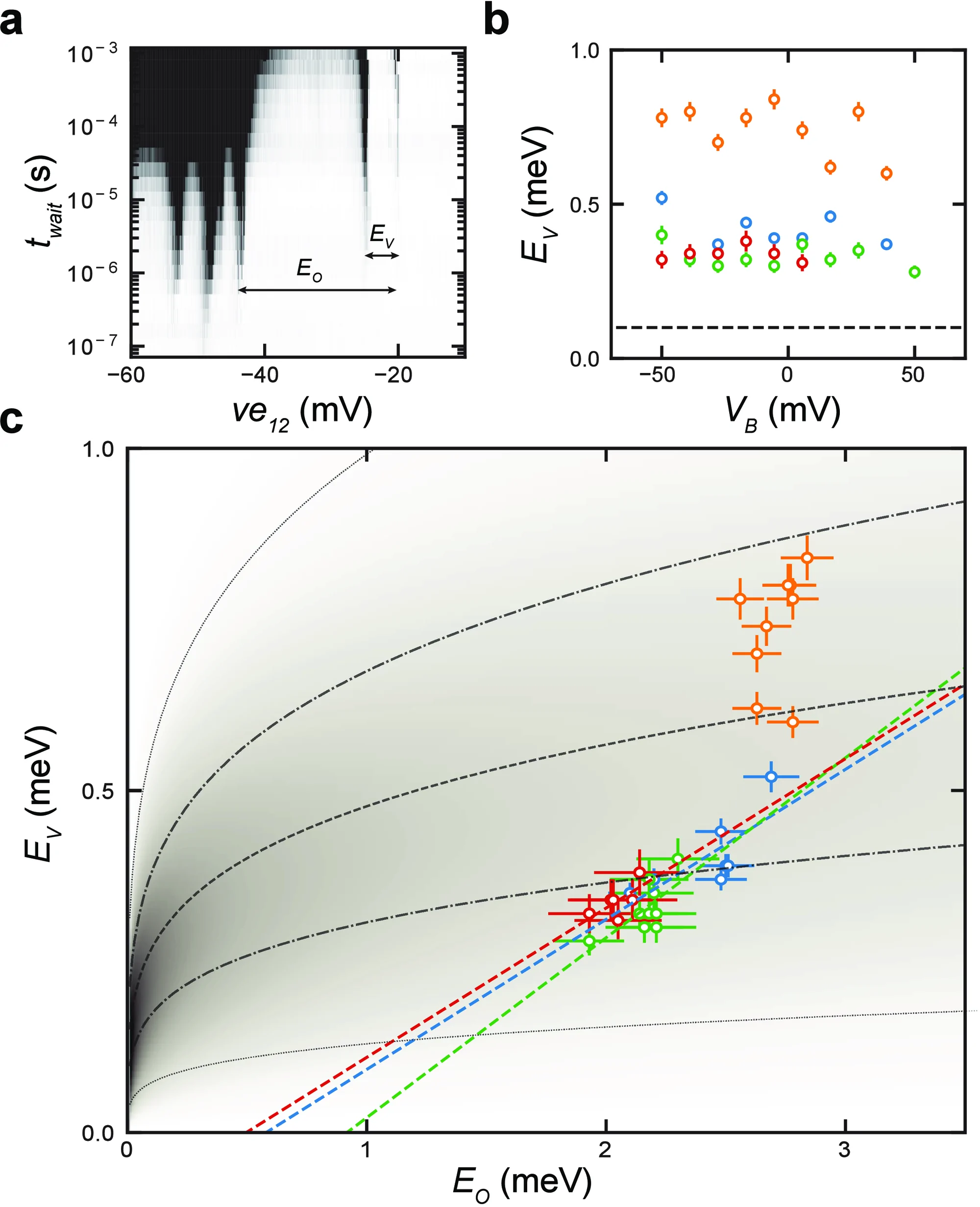
Single-electron spectroscopy. (a) Detuned axis pulsed spectroscopy (DAPS) maps of dot P1 obtained by sweeping the relative detuning (*ve*_12_) for different waiting times *t*_*wait*_. Charge tunneling at a specific detuning is enhanced by the presence of well-defined quantum states, giving rise to a faster relaxation rate. We extract *E*_*O*_ and *E*_*V*_ from the separation of the resonant peaks and convert them into energy using the specific lever arm for each gate. (b) Single-electron valley splitting (*E*_*V*_) extracted from DAPS for the four dots (P1 blue, P2 orange, P3 green, and P4 red) as a function of the voltage applied to the barriers (*V*_*B*_) between the dots. The dashed line is set at 0.1 meV, which is typically taken as the minimum required to ensure readout fidelities above 99%.^42^ (c) Cross correlation plot between *E*_*V*_ and *E*_*O*_. We fit the values from P1, P3, and P4, showing a high correlation with a linear function of the form *E*_*V*_ = α_*E*_*V*_/*E*_*O*__ *E*_*O*_ + *b*, from which we extract α_*E*_*V*_/*E*_*O*__ = 0.22(4) [eV/eV] and *b* = – 0.12(8) meV. The shaded area represents the theoretical probability density distribution for *E*_*V*_ as a function of *E*_*O*_, obtained by fitting the average z-y integral to the experimental data (see Supporting Information, Methods). The dark solid lines follow the median (dashed), 25th to 75th percentile (dot-dashed), and fifth to 95th percentile (dotted) of the theoretical valley splitting distributions.

We repeat the measurement for each dot in the array and for different confinement configurations, controlled by the barrier potential (*V*_*B*_) between the dots, to study the effect of electrostatic confinement on *E*_*V*_ and *E*_*O*_. Figure 2(b) shows the results of this analysis for the measured single-electron valley splitting for the four quantum dot array. Within the variability expected for the valley splitting,^12^ these quantum dots show consistently large values across a wide range of gate voltages, a necessary condition for high-fidelity operation during long pulse sequences implementing a quantum algorithm.

At the same time, we extract the first orbital energy from the DAPS spectrum for each confinement configuration. We plot the measured values of *E*_*V*_ and *E*_*O*_ in Figure 2(c) to analyze their correlation. We quantify the correlation between *E*_*O*_ and *E*_*V*_ using a Pearson correlation test, where 1 indicates a perfect correlation and 0 indicates no correlation, and use the corresponding p-value to assess the statistical significance of each test (see Supplementary Table S2). For the dots defined by P1, P3, and P4, we find correlation coefficients of ≈0.7, indicating a strong positive correlation between *E*_*V*_ and *E*_*O*_ and suggesting that confinement is a key aspect in driving the valley splitting to high values in these devices. This observation is consistent with the current theories of valley splitting in the random-alloy disorder-dominated regime and is expected for these heterostructures.^43^

To further characterize the relation between *E*_*O*_ and *E*_*V*_, we fit the data sets for P1, P3, and P4 with a linear relation of the form *E*_*V*_ = α_*E*_*V*_/*E*_*O*__ *E*_*O*_ + *b* (colored dashed lines in Figure 2(c)). The wave function of the dots with large *E*_*O*_, and therefore stronger electrostatic confinement, is strongly squeezed at the Si/SiGe interface and is expected to sample a larger distribution of Ge atoms in the SiGe spacer, enhancing the alloy-disorder coupling. The linear coefficient for each dot quantifies the local alloy-disorder landscape of the specific dot. We find, for all dots, a steep linear coefficient α_*E*_*V*_/*E*_*O*__ ≈ 0.20–0.24 meV/meV, indicating that electrostatic confinement can be an effective strategy for tuning the valley separation by hundreds of microelectronvolts. The linear trend between valley splitting and confinement is also measured in the 2DEG of similar heterostructures as a function of the out-of-plane magnetic field.^50^

We report the highest *E*_*V*_ within the array for the dot under P2, with an average *E*_*V*–*P*2_ = 0.76(2) meV and a low correlation coefficient of ≈0.3. These values set a new benchmark for single-electron valley splitting in buried quantum wells and are on par with the best values reported in SiMOS devices.^39^ The low correlation is generally unexpected for quantum dots defined in low-disorder heterostructures and could have multiple origins. On the one hand, the low correlation between *E*_*O*_ and *E*_*V*_ is consistent with a deterministic enhancement of the valley splitting beyond the alloy-disorder-dominated regime. On the other hand, it could be explained by the specific quantum dot pinning with a disorder center modifying the confinement and the valley coupling, besides the gate effects, and in line with the current theories,^26^ or by the location of the quantum dot on a node of the valley splitting oscillations, naturally less sensitive to changes in the confinement potential.^57^ While these measurements do not allow us to discern a specific origin, new techniques are currently being developed to better pinpoint the microscopic origin of valley-splitting.^58^

Next, we compare the experimental data with the theoretical predictions for single-electron quantum dots in these heterostructures.^43^ The theoretical distribution of the valley splitting depends on the electric field at the quantum-well interface (*E*_*Z*_), the microscopic Ge-alloy disorder, and the orbital energy.^43^ We estimate the electric field (*E*_*Z*_ ≈ 10 MV/m) from the maximum charge density in the two-dimensional electron gas.^55^ The alloy disorder enters the theoretical model when integrating over the z-coordinate and can be approximated phenomenologically as a constant that depends on the specific Ge concentration profile of the SiGe/Si/SiGe heterostructure, as determined by STEM (see Supporting Information). Contrary to the experimental results, the calculated valley-splitting distribution (see Supplementary Figure S12) predicts that most of our values should remain below 0.2 meV.

We then fit the disorder parameter to the experimental distribution of *E*_*V*_ to assess whether the theoretical model can predict the spread and dependence of the valley splitting as a function of orbital energy. We plot the distribution’s probability density as a function of orbital energy as a shaded area in Figure 2(c) and highlight its evolution as a function of the orbital energy by plotting the median (dashed line), 25th to 75th percentile (dot-dashed line), and 5th to 95th percentile (dotted) of the theoretical distributions. As expected, the spread of the valley-splitting distribution increases with its average and with the orbital energy^42^ and matches the observed spread in the experimental data.

We conclude the characterization by measuring two-electron singlet–triplet energy splitting via magnetospectroscopy (*E*_*ST*_) and Pauli spin blockade spectroscopy (*E*_*PSB*_). *E*_*ST*_ is the relevant energy scale for the spin-to-charge conversion readout using the Pauli exclusion principle^29,59^ and therefore sets an ultimate limit to the possible readout fidelity and operation temperature of electron spin qubits in Si/SiGe.

Figure 3(a) shows a typical magnetospectroscopy map across the 1e to 2e transition under the virtual plunger vP1 as a function of the in-plane magnetic field (B). The thick black line is a fit of the transition line to the theoretical model (see Supporting Information, Methods) from which we estimate the singlet–triplet splitting *E*_*ST*_ = *g μ*_*B*_ *B*_*ST*_, where *g* = 2 is the electron g-factor, μ_*B*_ is the Bohr magneton, and *B*_*ST*_ corresponds to the magnetic field at which the final two electron state transition from a singlet (*S*_0_) to a triplet (*T*_–_) spin state, i.e., the position of the kink in Figure 3(a). For the external dots P1 and P4, we perform magnetospectroscopy of the addition line from the reservoir (1e → 2e) and the interdot transition ((1,1) → (2,0)) and find comparable values of *E*_*ST*_. From the fit to the magnetospectroscopy trace, we also extract the lever arm of each virtual plunger gate in agreement with the values previously measured via temperature activation.

**Figure 3:**
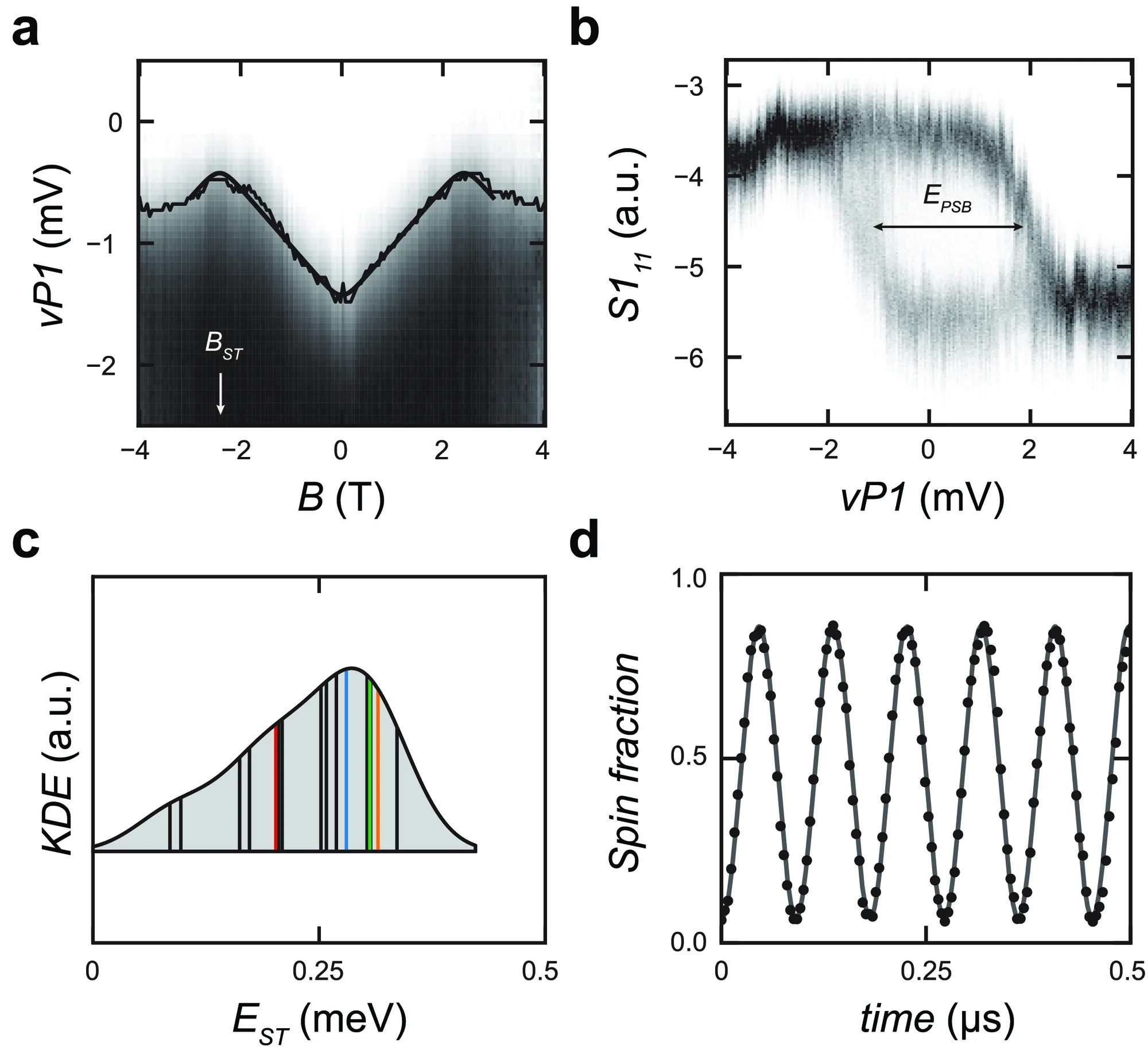
Two-electron spectroscopy. (a) Magnetospectroscopy of the second-electron addition line under vP1 as a function of the external in-plane magnetic field *B*. The thick dark line is a fit of the transition line to the theoretical model from which we extract the position of the kink at *B*_*ST*_ = 2.4(1) T, corresponding to a singlet–triplet splitting of *E*_*ST*_ = 0.28(1) meV, and a lever arm of α = 0.11 eV/V in agreement with the previous measurement via temperature activation. (b) Histogram of the reflected signal amplitude from the sensing dot S1 for 50,000 single-shot measurements as a function of vP1 across the (1,1)–(0,2) anticrossing from which we extract the two-electron singlet–triplet splitting from Pauli spin blockade spectroscopy (*E*_*PSB*_). (c) Kernel density estimation (KDE) of the probability density for the singlet–triplet splitting extracted from 17 lithographically identical quantum dots from four different devices and two different wafers (nine values from ref 30). The solid lines within the distribution represent the single instances of *E*_*ST*_ for each dot; the colored lines correspond to the four dots (P1–P4). From the distribution, we extract a mean singlet–triplet splitting of *E̅*_*ST*_ = 0.24(7) (meV). (d) Spin fraction for the double dot under P1 and P2 as a function of the waiting time at the symmetry point of the (1,1) charge state, showing coherent oscillation between singlet and triplet states mediated by the exchange and used to validate the spin selectivity of the readout scheme before Pauli spin blockade spectroscopy.

We collect singlet–triplet energy splitting from 17 lithographically identical quantum dots from four different devices and two nominally identical wafers (nine values from ref 30) and plot in Figure 3(c) the kernel density estimation (KDE) of the probability density for *E*_*ST*_. The black lines within the distribution represent the individual instances of singlet–triplet splitting, while the colored lines highlight the instances from the four quantum dots P1 to P4. Contrary to the Rayleigh or Rice-like distribution expected for the single-electron valley splitting in the alloy disorder-dominated regime, the measured distribution for *E*_*ST*_ shows a bell-like shape with an average singlet–triplet splitting of *E*_*ST*_ = 0.24(7) meV. Notably, in all dots, *E*_*ST*_ is substantially lower than the measured *E*_*V*_. This suggests that the formation of Wigner molecule states^60–62^ —in which a strong Coulomb repulsion spatially localizes the two electrons—limits the singlet–triplet splitting rather than single-electron valley splitting.

Finally, we corroborate the characterization of two-electron states by performing Pauli spin blockade spectroscopy across the (1,1) → (2,0) transition. In this charge configuration, the singlet–triplet splitting is limited by the valley energies and should provide a precise hallmark in the case of low occurrences of valley splitting.^29^ We perform initialization via readout and postselection on shots with the same initial state. By scanning the interdot transition as a function of the plunger gate, the sensor signal splits, and a window opens in which singlet and triplet states are selectively associated with different charge states, known as the PSB window. We report the results in Figure 3(b). We use the lever arm to convert the width of the PSB window and extract an estimation for the singlet–triplet splitting of *E*_*PSB*_ ≈ 0.42(2) meV. We repeat the measurement for the four quantum dots and measure coherent oscillations of the spin fraction after each sequence, as in Figure 3(d), to validate the spin-selectivity of the readout scheme (see Supporting Information).

We find that the two-electron *E*_*PSB*_ energy splittings are in quantitative agreement with magnetospectroscopy measurements for the internal dots P2 and P3 and are lower than the single-electron *E*_*V*_ values for the respective dots, similar to what was reported in ref 63. For the external dots, P1 and P4, we find a larger *E*_*PSB*_ value than *E*_*ST*_. This can be explained by the different tuning conditions of the two measurement regimes. We perform PSB spectroscopy in the so-called isolated mode, i.e., under low tunnel coupling between the sensors and the external dots. As a result, the confinement of the external dots is tighter during PSB spectroscopy, leading to higher singlet–triplet energy levels, consistent with the previous interpretation that *E*_*S*_ *T* is limited by the confinement and the formation of Wigner molecule states.

We summarize the measurements of single and two-electron states in buried Si quantum wells in Figure 4. We report the charging energy (*E*_*C*_), single electron orbital energy (*E*_*O*_), single electron valley splitting (*E*_*V*_), two-electron singlet–triplet splitting from magnetospectroscopy (*E*_*ST*_), and Pauli spin blockade spectroscopy (*E*_*PSB*_). As expected, we find that *E*_*V*_ and *E*_*ST*_ constitute the lowest excitations, followed by *E*_*O*_ and *E*_*C*_. We report consistently high values of valley splitting, with the highest of 0.76(2) meV, setting a new benchmark for electrons in buried quantum wells and comparable to the best values in SiMOS. We find a strong correlation between orbital and valley splitting with a large proportionality coefficient of 0.22 eV/eV, indicating that confinement can be effectively used in low-disorder heterostructures to tune and enhance valley splitting. We extend our statistics on *E*_*ST*_ and confirm an average of 0.24(7) meV across 17 lithographically identical quantum dots, over four different devices and two wafers.

**Figure 4:**
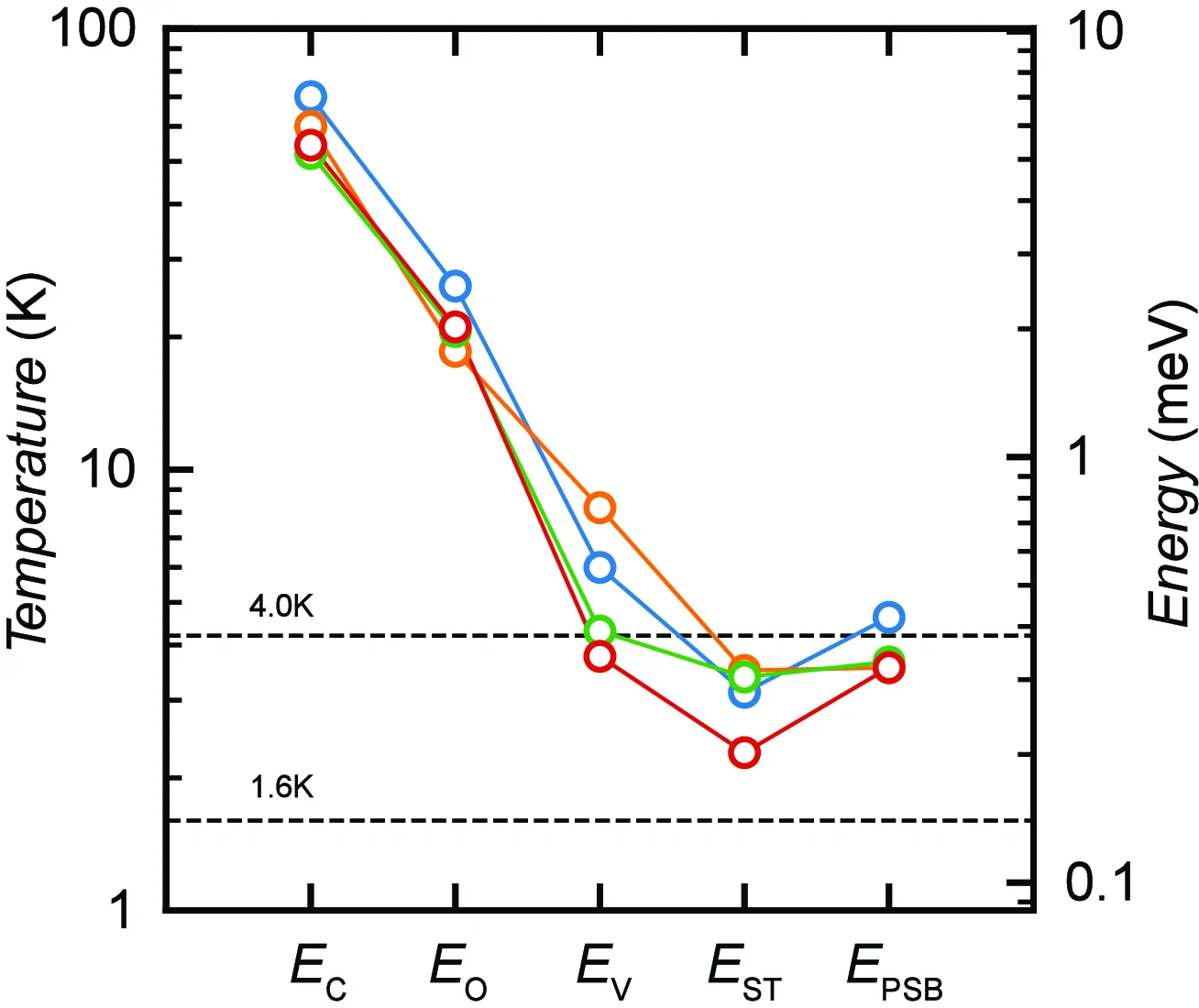
Single-electron and two-electron energy states in silicon quantum dots. We report the charging energy (*E*_*C*_), single electron orbital energy (*E*_*O*_), single electron valley splitting (*E*_*V*_), two-electrons singlet–triplet splitting measured via magnetospectroscopy (*E*_*ST*_), and Pauli spin blockade spectroscopy (*E*_*PSB*_) for all the four dots (P1 blue, P2 orange, P3 green, and P4 red). The dashed lines correspond to the operation temperature of a closed-loop ^4^He refrigerator (1.6 K) and of a liquid ^4^He bath (4.2 K).

The comparison with existing alloy-disorder models further delineates their range of validity, highlighting the need for theoretical descriptions that capture the strong confinement, low-disorder regime explored here. Conversely, direct structural characterization of measured devices by atom probe tomography^42^ as well as synchrotron X-ray nanodiffraction to map strain and compositional fluctuations^64^ will be crucial to provide most accurate microscopic inputs these models require. While we cannot pinpoint a specific microscopic origin for the large values reported here, we highlight several features that allow our heterostructures and gate stacks to define a strong electrostatic confinement contributing to driving the valley splitting, such as the shallowness of the quantum well, the use of thin high-k dielectrics in the gate stack, and the aggressive gate pitch. All of these considerations build a practical path to enhancing valley splitting in low-disorder heterostructures. Furthermore, new methodologies are currently being developed to identify the microscopic mechanism driving the valley splitting.^58^ We envision that the further characterization of these low-disorder quantum wells with such techniques and the comparison with heterostructures where the alloy disorder is intentionally enhanced^48,50,51^ will provide ultimate insight into the microscopic physics and chart a practical path toward engineering buried quantum wells into a platform that uniquely combines well-defined energy states, low electric and magnetic disorder, and compatibility with advanced semiconductor manufacturing processes.

## Supplementary Material



## Data Availability

All the code used to derive the figures and analyze the data is included with the data and is available at the 4TU.ResearchData international data repository at 10.4121/6bea90c0-1801-4aaf-a052-07c0dbbd0920.v1

## Notes

Additional SEM and AFM of quantum dot devices, additional TEMs and EDS of the quantum dot and heterostructure stacks, read-out reflectometry characterization, additional charge stability diagrams, extended virtualization matrix, extended lever arm measurements, extended magnetospectroscopy for devices A and B, extended pulsed spectroscopy, extended DAPS measurements, extended PSB spectroscopy, extended coherent singlet triplet oscillations, supplementary simulations of the valley splitting distributions, supplementary simulations of the valley splitting distributions in the presence of a small Ge concentration in the quantum well, summary table of the single and two electron energy scales, and summary table of Pearson correlation test for *E*_*V*_ vs *E*_*O*_ (PDF)
