# Supplementary material for: Confinement Drives Valley Splitting above 4 K in Buried Silicon Quantum Wells
Source: Nano Lett. 2026 Oct 22;26(43):15131–9. doi: 10.1021/acs.nanolett.6c04172 (PMC13741925; doi:10.1021/acs.nanolett.6c04172)
Supplement: Supplementary file 1 [file nl6c04172_si_001.pdf]

Supplementary Information: Confinement drives valley splitting above 4K in buried silicon quantum wells

Davide Degli Esposti,[*,†] Emma C. Brann,[‡] Asser Elsayed,[†] Davide Costa,[†] Mark Friesen,[‡] and Giordano Scappucci[*,†]

†*QuTech and Kavli Institute of Nanoscience, Delft University of Technology, Lorentzweg 1, 2628 CJ Delft, The Netherlands*

‡*Department of Physics, University of Wisconsin-Madison, Madison, Wisconsin, 53706, United States*

E-mail: d.degliesposti@tudelft.nl; g.scappucci@tudelft.nl

Methods

Si/SiGe heterostructure growth. The ^{28}Si/SiGe heterostructures are grown on a 100-mm n-type Si(100) substrate using an Epsilon 2000 (ASMI) reduced-pressure chemical vapor deposition reactor. The reactor is equipped with a $^{28}SiH_4$ gas cylinder (1% dilution in H_2) for the growth of isotopically enriched ^{28}Si with 800 ppm of residuals of other isotopes[1]. Starting from a Si substrate, the layer sequence of all heterostructures comprises a step-graded $Si_{(1-x)}Ge_x$ layer with a final Ge concentration of $x = 0.31$ achieved in four grading steps ($x = 0.07$, 0.14, 0.21, and 0.31), followed by a $Si_{0.69}Ge_{0.31}$ strain-relaxed buffer (SRB). The step-graded buffer and the SRB are ≈3 μm and ≈2.4 μm thick, respectively. After the SRB, we grow a tensile-strained ^{28}Si quantum well conformal to the substrate and thickness of 6.9 nm, nominally identical to[2]. The heterostructure is then terminated with a 30 nm-thick SiGe spacer, grown under the same conditions as the virtual substrate. The surface of the SiGe spacer is passivated with DCS at 500 °Cbefore exposure to air[3]. We confirm the Ge concentration in the spacer and virtual substrate via secondary ion mass spectrometry (similar to Fig.S13 from ref.[4]) and quantitative electron energy loss spectroscopy. These heterostructures demonstrate very low-disorder quantum wells characterized by a mobility of the two-dimensional electron gas exceeding 3×10^5 cm^2/Vs[2]. From an electrical perspective, the absence of spurious dots in this experiment and in many others[5–7] employing nominally identical heterostructures suggests that Ge incorporation or diffusion into the well is highly unlikely. Further, we exclude Ge incorporation into the ^{28}Si quantum well based on structural characterization of heterostructures grown with an identical protocol in Ref.[4]: Secondary Ion Mass Spectroscopy on thicker control quantum wells to overcome the knock-on artifact places an upper bound of 0.02% Ge in the ^{28}Si layer, while Atom Probe Tomography confirms sharp interfaces ($4\tau = 0.79 \pm 0.31$ nm) and flat isoconcentration surfaces (RMS roughness < 0.15 nm), together ruling out any significant Ge diffusion into the quantum well within the approximate 5 nm separating the two Si/SiGe interfacial region. Further characterisation by Electron-Energy-Loss-Spectroscopy (EELS) of the devices is provided in

Supplementary Fig.S2.

Device fabrication. The fabrication process for the quantum dot spin qubit devices comprises: reactive ion etching of mesa-trench and pre-markers; selective P-ion implantation and activation by rapid thermal annealing at 700 °C; atomic layer deposition (ALD) of a 10-nm-thick Al_2O_3 gate oxide; sputtering of Al gate; selective chemical etching of the dielectric with BOE (7:1) followed by electron beam evaporation of Ti:Pt to create ohmic contacts. All patterning is performed by optical lithography at the four-inch wafer scale. Single and multi-layer quantum dot devices are fabricated on wafer coupons from the same H-FET fabrication run and share the process steps listed above. Single-layer quantum devices feature all the gates in a single evaporation of Ti:Pd (3:17 nm), followed by the deposition via ALD of a 5 nm thick AlOx layer and consequent evaporation of a global top screening gate of Ti:Pd (3:27 nm). Multi-layer quantum dot devices feature three overlapping gate metallizations with increasing Ti:Pd thicknesses (3:17 nm, 3:27 nm, 3:27 nm), each isolated by a 5 nm-thick AlOx dielectric. Finally, a 5 nm AlOx layer separates the gate stack from the micro-magnets (Ti:Co, 5:200 nm). All patterning in quantum dot devices is performed using electron-beam lithography.

Measurement setup and cryogenics. All the experiments are performed in a Blufors LD400 dilution refrigerator at a fixed temperature of 150 mK to reduce heating effects[8]. The quantum dot device is wire-bonded to a custom design PCB featuring on-chip bias Tees (RC time ≈ 100 ms) to sum the DC and RF voltages. The DC voltages are supplied by a battery-powered, home-built DAC module (D5a), while the baseband control and readout pulses are generated with a series of QCM and QRM modules embedded in a QBlox Cluster. The readout of the two charge sensors is performed via frequency multiplexing by connecting each single-electron transistor to a tank circuit with a different resonant frequency (see Supplementary Fig.S3). The reflected signal is then amplified at the 4 K stage by a Cosmic Microwave Technologies CMT-BA1 cryogenic amplifier (≈ 30 dB) and further amplified at room temperature (≈ 40 dB) to match the 1 V dynamic range of the digitizer module.

Lever arm estimation. We extract the lever arm of each virtual gate by measuring the first-electron-addition line as a function of the mixing chamber temperature (T_{MC}). We tune the tunnel coupling to the regime where the broadening of the first addition line is limited by temperature broadening. In this regime, the signal (S_i) of the first polarization line can be expressed as:

$$A + B + C \tanh \alpha(V - V_0)\beta$$

where A, B, and α are fitting parameters and $\beta = \frac{e}{2k_B T_e}$ with k_B the Boltzmann constant and T_e the electron temperature. For sufficiently high temperatures and good thermalization, the electron temperature can be approximated with the temperature of the mixing chamber ($T_e \approx T_{MC}$) We find that the condition is satisfied for $T_{MC} > 0.5$ K. We validate this approximation by verifying that the extracted lever arm is constant in this regime, repeating the measurement at multiple temperatures between 0.5 and 1 K. We repeat the measurement for each dot, using either nearby charge sensors or nearby dots as electron reservoirs.

Pulsed spectroscopy. We perform pulsed spectroscopy of external quantum dots directly coupled to multi-electron charge sensors that act as electron reservoirs. We sweep the virtual plunger voltage vP_i across the first electron addition line as a function of the amplitude of a superimposed square pulse of fixed frequency, typically of $f = 25$ kHz. We tune the tunnel coupling to be low compared to the frequency of the superimposed square wave, such that the electron tunneling to the dot is most sensitive to the presence of well-defined single-electron states. We record the tunneling by measuring the state of the nearby charge sensor for integration times (t_{int}) much longer than the period of the superimposed square wave ($t_{int} \approx 1 - 10$ ms $\gg 1/f$).

Detuning axis pulsed spectroscopy. We perform detuning axis pulsed spectroscopy (DAPS) by pulsing the virtual detuning ($ve_{ij} = P_i - P_j$) between two quantum dots in a single-shot experiment and averaging the signal over multiple shots. We initialize the double dot in the (1,0) charge configuration by pulsing at the 'init' point deep in detuning in the (1,0) configuration and waiting for a long time, typically greater than 100 μs. Next, we

acquire the reference signal at the 'zero' point near the (1,0) to (0,1) charge transition by integrating the charge sensor for 5 μs. We then quickly pulse diabatically across the charge anticrossing at the tunneling point by sweeping the detuning ve_{ij} inside the (0,1) charge state for different waiting times (t_{wait}). Finally, we pulse back to the 'zero' to acquire the measurement signal. We record the difference between the reference and measurement signals to filter out low-frequency charge fluctuations in the charge sensor and quantum dot device. We tune the tunnel coupling to be lower than the waiting time used in the measurement, thereby enhancing the contrast of resonant tunneling.

Magnetospectroscopy. We perform magnetospectroscopy experiments in quantum dot devices cooled in a dilution refrigerator with a base temperature of $T_{\mathrm{MC}} \approx 10$ mK. We use devices lithographically similar to those described in ref.[9]. We tune the quantum dots in the single-electron regime to isolate the 1e $\rightarrow$ 2e transition. We start the magnetospectroscopy measurement from the quantum dot closest to the sensing dot, using the remaining dots as an electron reservoir. We use the impedance of a nearby sensing dot to monitor the charge state of every quantum dot. The impedance of the sensing dot is measured using RF reflectometry. The signal is measured by monitoring the reflected amplitude of the RF readout signal through a nearby charge sensor. We fit the 1e $\rightarrow$ 2e transition as a function of the magnetic field with the relation[4,10]:

$$V_{\mathrm{P}} = \frac{1}{\alpha\beta_{\mathrm{e}}} \ln \frac{e^{\frac{1}{2}kB+\beta_{\mathrm{e}}E_{\mathrm{ST}}}(e^{kB}+1)}{e^{kB}+e^{2kB}+e^{kB+\beta_{\mathrm{e}}E_{\mathrm{ST}}}+1} \tag{S1}$$

where α is the lever arm, V_{P} is the plunger gate voltage, E_{ST} is the singlet-triplet energy splitting, $k = g\mu_{\mathrm{B}}\beta_e$, $\beta_e = 1/k_{\mathrm{B}}T_{\mathrm{e}}$, $g = 2$ is the g-factor in silicon, μ_{B} is the Bohr magneton, $\mathbf{B}$ is the magnetic field, k_{B} is Boltzmann's constant, and T_{e} is the electron temperature. E_{ST} is linked to the position of the kink ($\mathbf{B}_{\mathrm{ST}}$) in the magnetospectroscopy traces by the relation $E_{\mathrm{ST}} = g\mu_{\mathrm{B}}\mathbf{B}_{\mathrm{ST}}$.

Valley splitting. In the disorder-dominated regime, the intervalley coupling Δ is dis-

tributed about zero with variance[11]:

$$\sigma_\Delta^2 = \frac{a_0^3}{16}\left(\frac{\Delta E_c}{X_w - X_s}\right)^2 \int dz|\psi_{env}(z)|^4 \bar{X}(z)(1-\bar{X}(z))\times \int dxdy|\psi(x,y)|^4 \tag{S2}$$

where $a_0 = 0.543$ nm is the width of the conventional cubic unit cell, ΔE_c is the conduction band offset between the barrier region and the well, and X_s, X_w are the concentrations of Si deep in the well and barrier region, respectively.

Because the z part of the integral describes alloy disorder averaged over the device, we can approximate it as constant at all measurement points. We may also approximate the y orbital splitting as constant by absorbing the y piece of the orbital integral into this constant. Then, variance can be estimated for some E_{OS} by[12]:

$$\sigma_\Delta^2 \approx \eta_{yz} \int dx|\psi(x;E_{OS})|^4 \tag{S3}$$

We estimate the constant η_{yz} using an averaged valley splitting and orbital splitting values across this device:

$$\eta_{yz} = \frac{\overline{\sigma}_\Delta^2}{\int dx|\psi(x;\overline{E}_{OS})|^4} \approx 4.12 \text{ meV}^2 \cdot \text{nm} \tag{S4}$$

$\overline{\sigma}_\Delta^2$ is computed using maximum likelihood approximation to fit a Rayleigh distribution to the measured values of E_{VS}.

Extended Figures

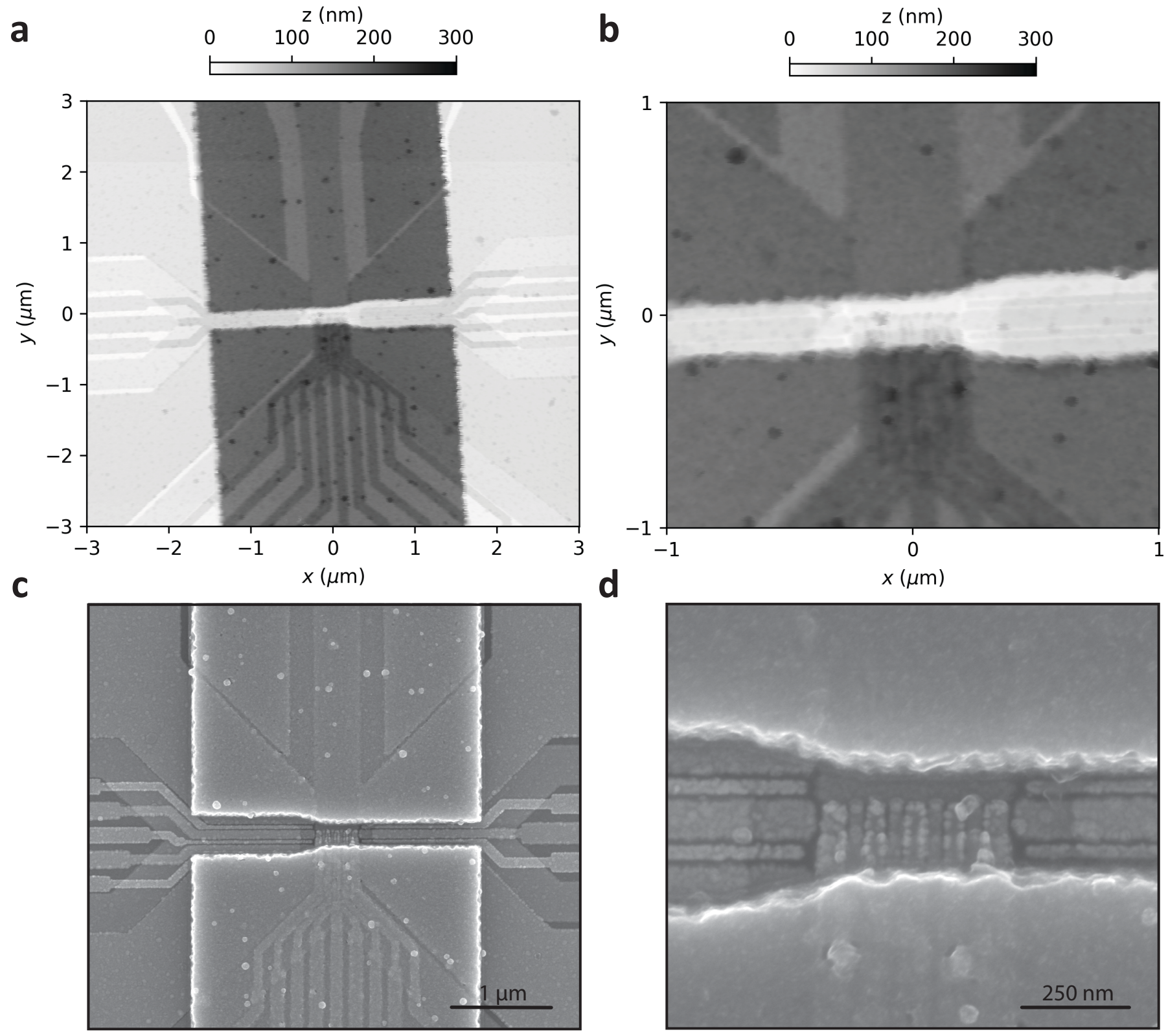

Figure S1: **AFM and SEM characterization.** (a-b) Atomic force microscopy (AFM) images of a lithographically identical device to the one used in the main text. The gate layout and fanout are visible as morphology beneath the two large rectangles around the quantum dot plungers that form the on-chip micro-Magnet. (c-d) Scanning Electron Microscopy (SEM) images of the same device as in (a-b).

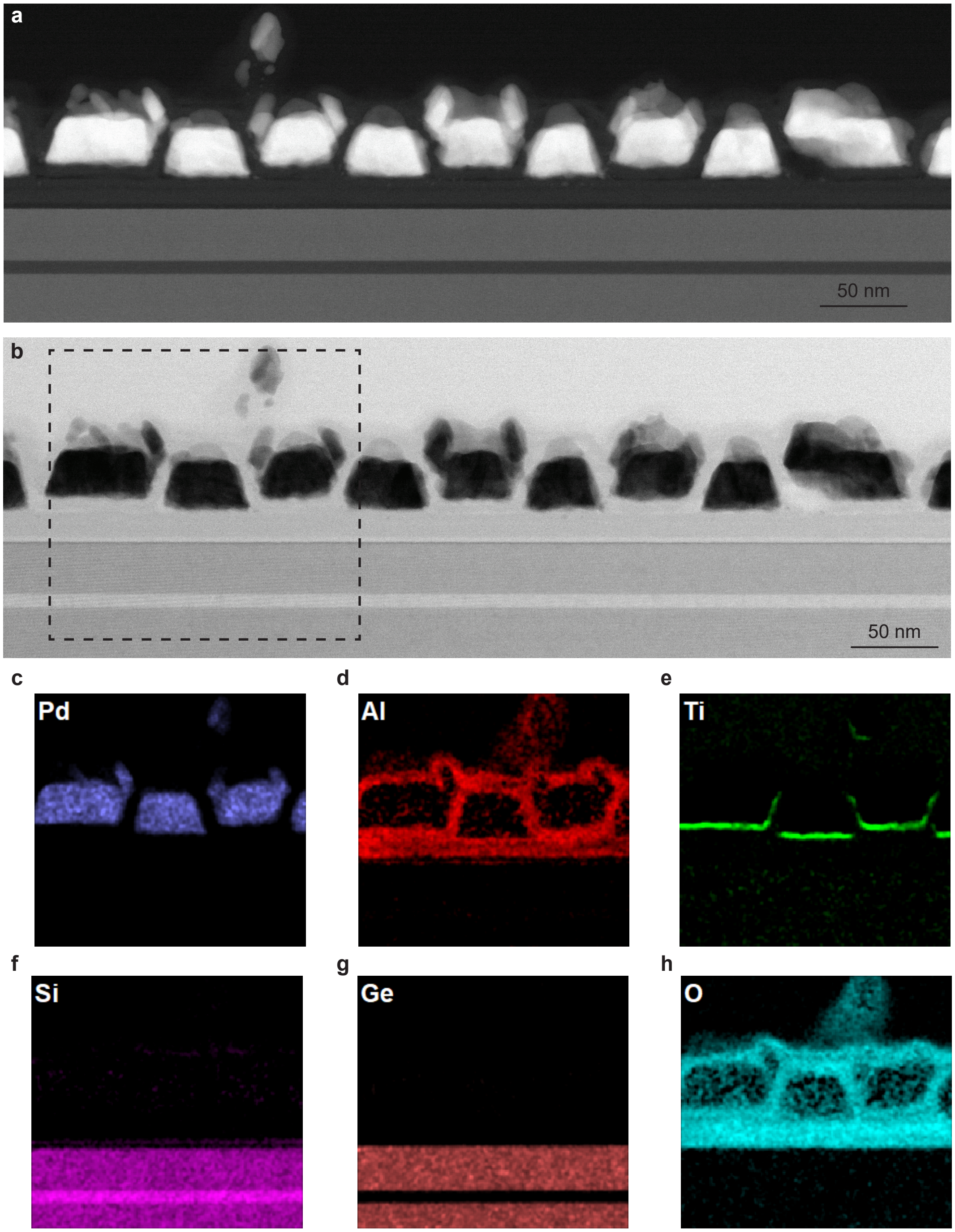

Figure S2: **STEM and EELS characterization.** (a-b) High-resolution high-angle aberration corrected (HAADF) and bright field (BF) scanning transmission electron microscopy (STEM) images of a four quantum dot device lithographically identical to the device characterized in the main text and the same device of Fig.S1. (c-h) Electron energy loss spectroscopy (EELS) maps of the region highlighted in panel (b) for the Pd, Al, Ti, Si, Ge, and O composition.

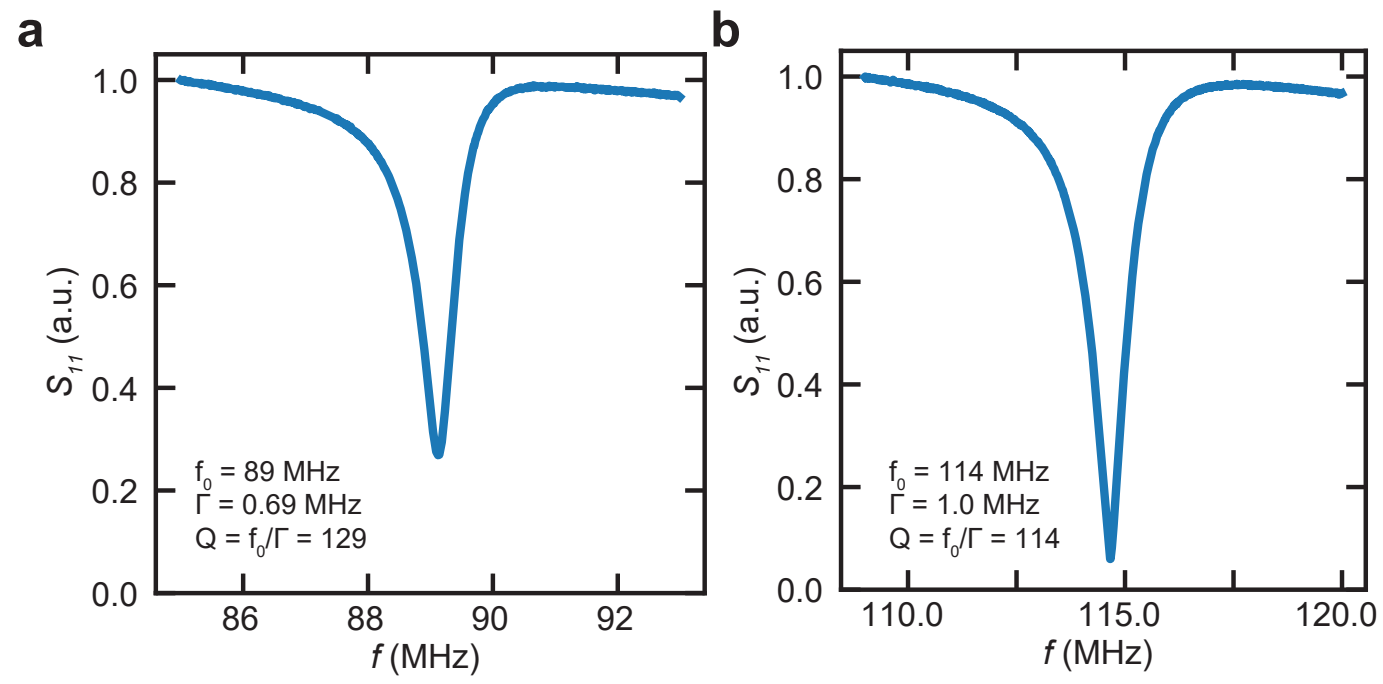

Figure S3: **Reflectometry LC-tank circuits.** (a-b) Amplitude of the reflected signal (S_{11}) as a function of frequency (f) used to identify the resonances of the two LC tank circuits connected to the accumulation gates of the two sensing dots S1 (a) and S2 (b). The LC-tank circuits are formed by two high kinetic inductance superconducting resonators (nominal inductance of ≈ 5 μH and ≈ 3 μH) and by the parasitic capacitance to ground (≈ 1 pF) of the printed circuit board (PCB), bond wire, and sample. We report the resonance frequency (f_0), full-width-half-maximum (Γ) and quality factor ($Q = f_0/\Gamma$)

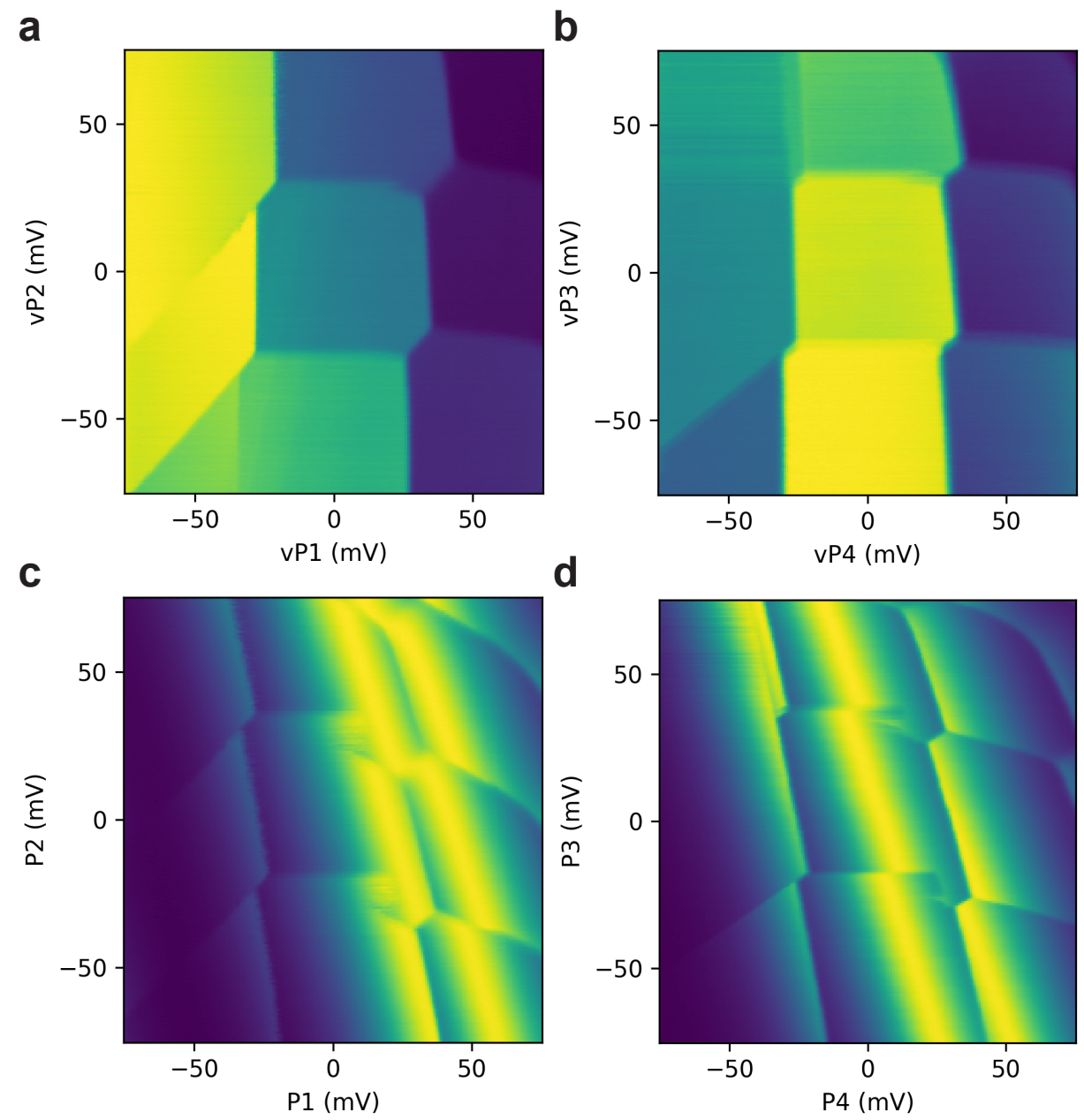

Figure S4: **Charge stability diagrams.** (a-b) Charge stability diagram for the four virtualized plungers (vP1-vP2 and vP3-vP4) centered around the (1,1) charge state. (c-d) Charge stability diagram for the four real plungers (P1-P2 and P3-P4) centered around the same configuration as (a-b). Each double-dot pair is sensed by the nearby charge sensor: S1 for P1-P2 and S2 for P3-P4.

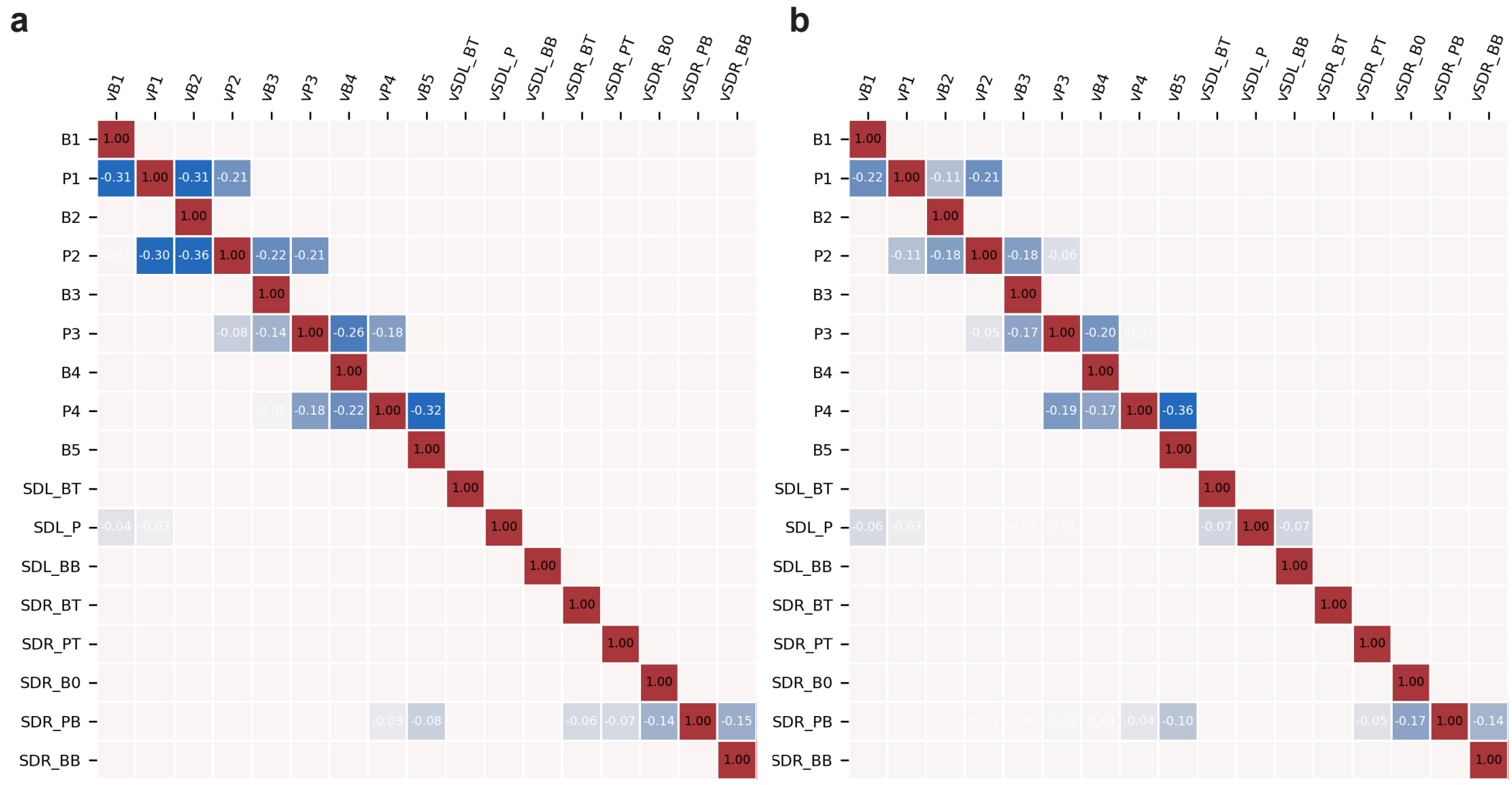

Figure S5: **Virtual gate matrix.** Virtualization matrices for the device analyzed in the main text (a) and for a lithographically identical device (b). The virtual gates (vP_i) are defined as a linear combination of the real gates (P_j) with coefficients given by the virtualization matrix ($M_{i,j}$). In the white squares, the specific virtual matrix coefficient is zero.

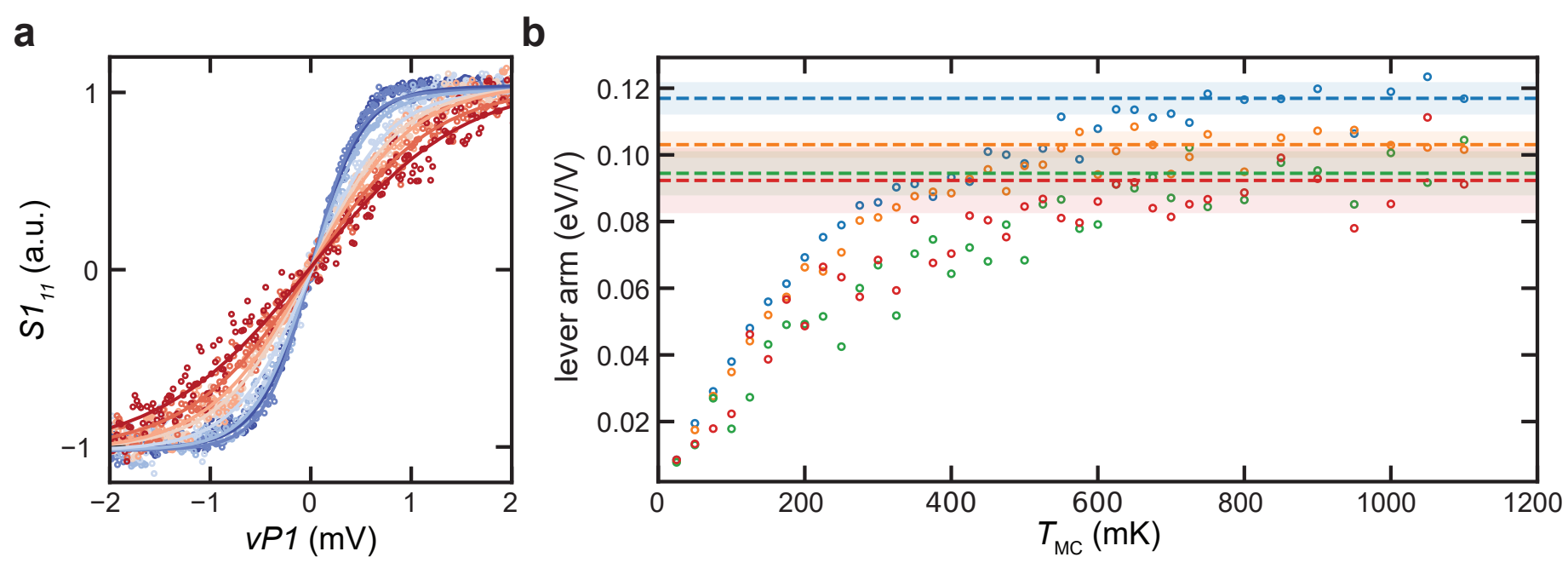

Figure S6: **Lever arm characterization.** (a) First electron addition line under vP1 (dots) for multiple temperatures of the mixing chamber between 25 mK and 1100 mK, with superimposed fit (solid line) used to extract the lever arm. We extract the lever arm α by fitting the addition line to the formula $A + B + C \tanh \alpha(V - V_0)\beta$ where A, B, and α are fitting parameters and $\beta = \frac{e}{2k_B T_{mc}}$. (b)Fitted lever arm as a function of the mixing chamber temperature (T_{MC}). We find that the fits converge to a fixed value for temperatures $T_{MC} > 500$ mK when the broadening of the addition line is dominated by the temperature. We extract the lever arm for each dot as the average in this regime (dashed line) and estimate the uncertainty as the standard deviation (shaded area).

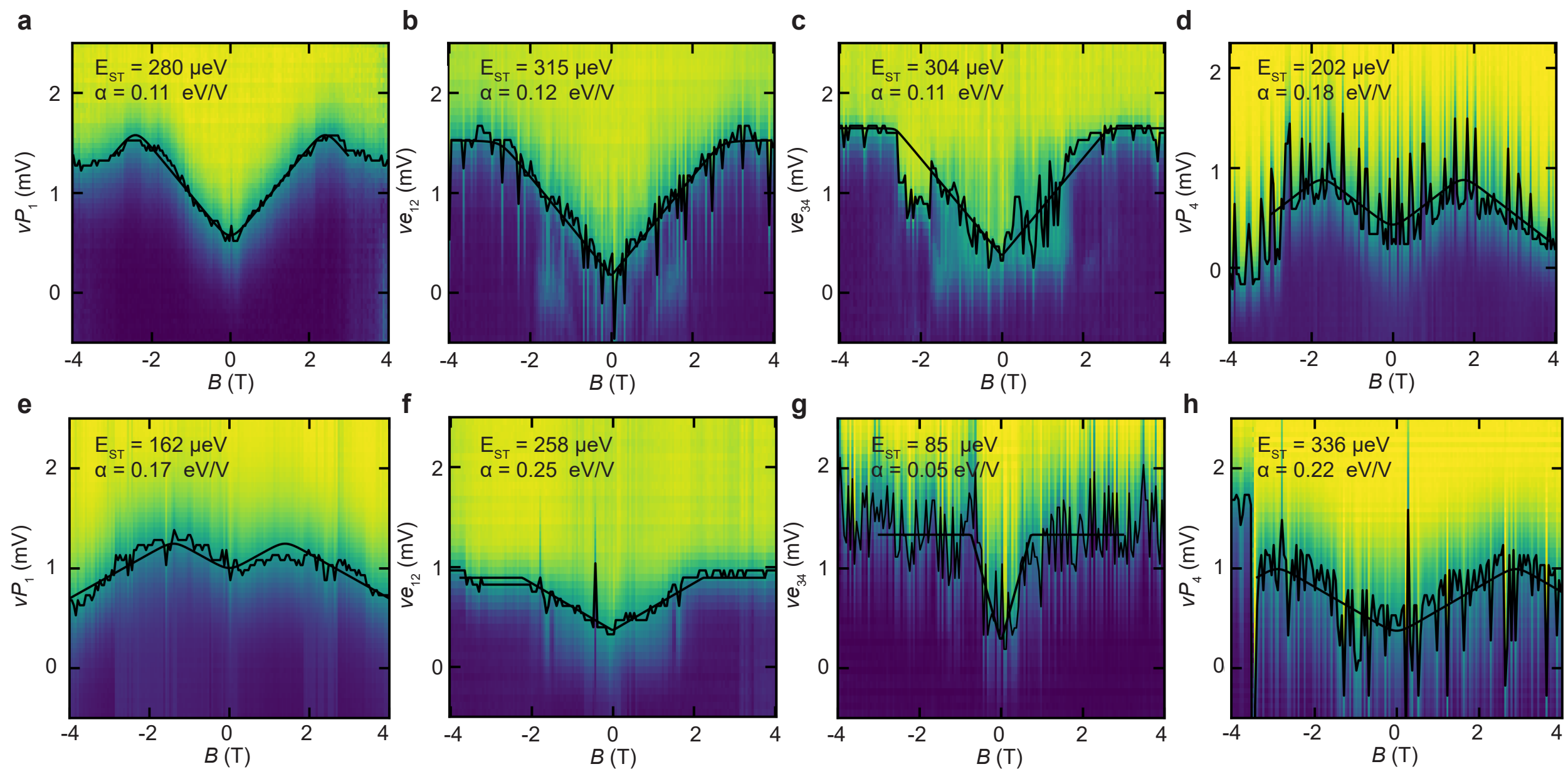

Figure S7: **Extended - Magnetospectroscopy.** Complete magnetospectroscopic characterization of the two four-dot devices, from (a) to (d) the device of the main text and from (e) to (h) a lithographically identical device. We fit the second electron addition line to the theoretical model (see Methods) and extract the Singlet-Triplet energy splitting (E_{ST}) and the lever arm (α) reported for each quantum dot.

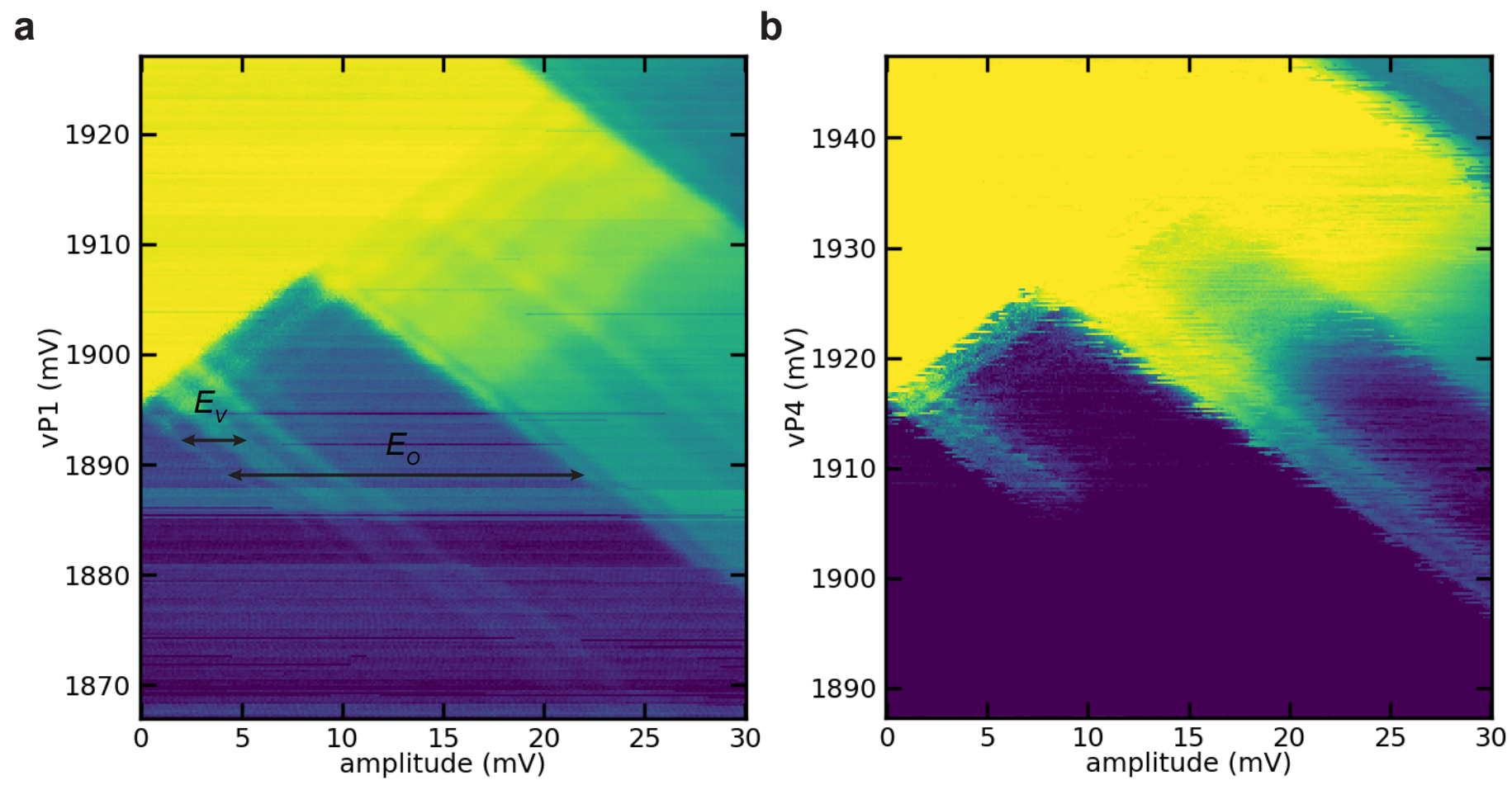

Figure S8: **Pulsed-spectroscopy.** Pulsed spectroscopy characterization of the external dots (vP1 and vP4) of the main device (device A) directly coupled to the sensing dots. We scan the plunger voltage (vP) across the first electron addition line as a function of the amplitude of a square pulse applied to the same gate (see Methods). From this measurement, we extract a single-electron valley splitting of 0.38(3) meV (0.31(2) meV) for P1 (P4) and correspondingly an orbital energy of 2.1(1) meV (1.4(1) meV).

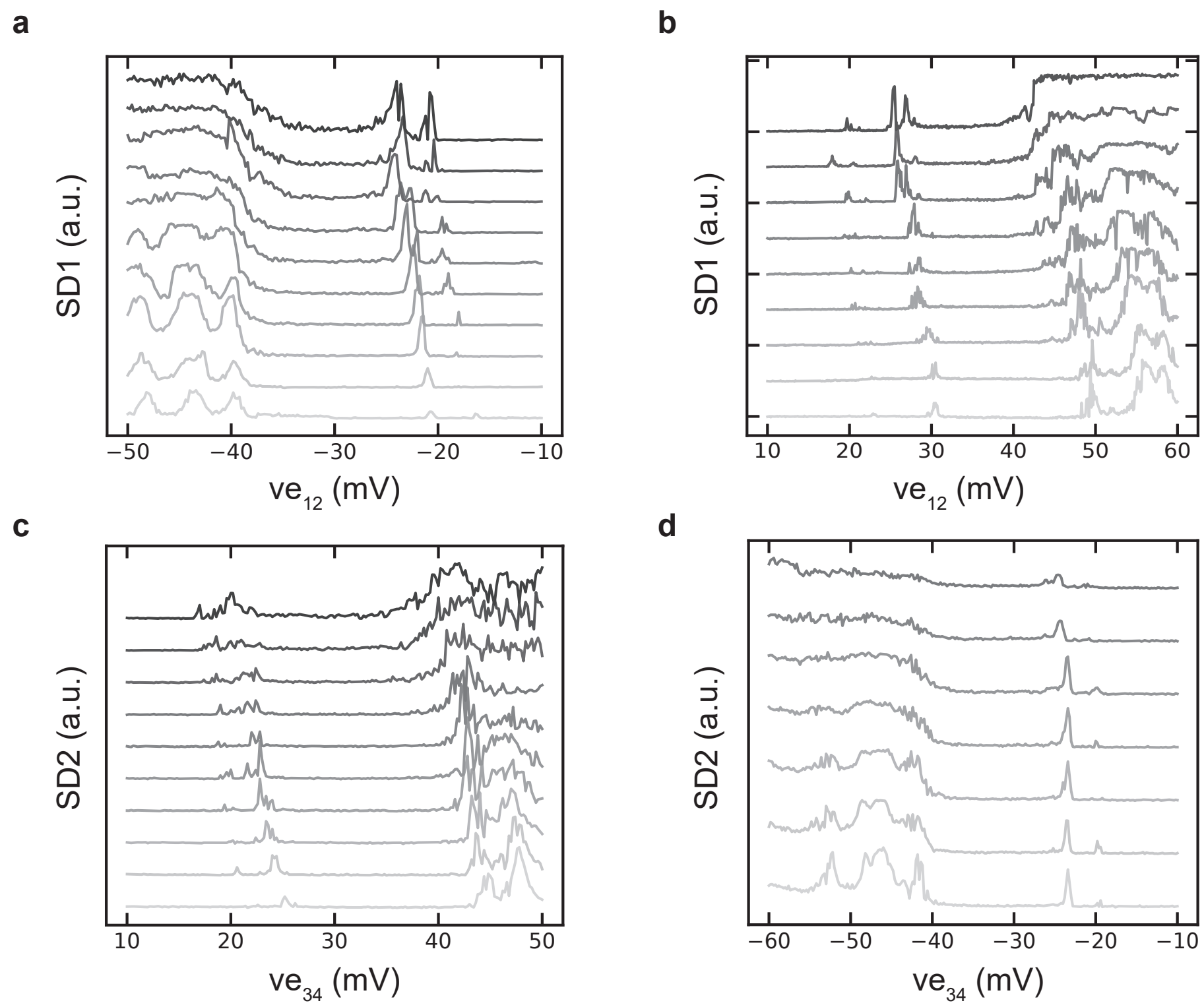

Figure S9: **Extended - Detuning Axis Pulsed Spectroscopy (DAPS).** DAPS traces for the four quantum dots (P1 to P4, from (a) to (b)) as a function of the voltage applied to the two interdot barriers (B2 and B4). Measurements are offset on the vertical axis for clarity.

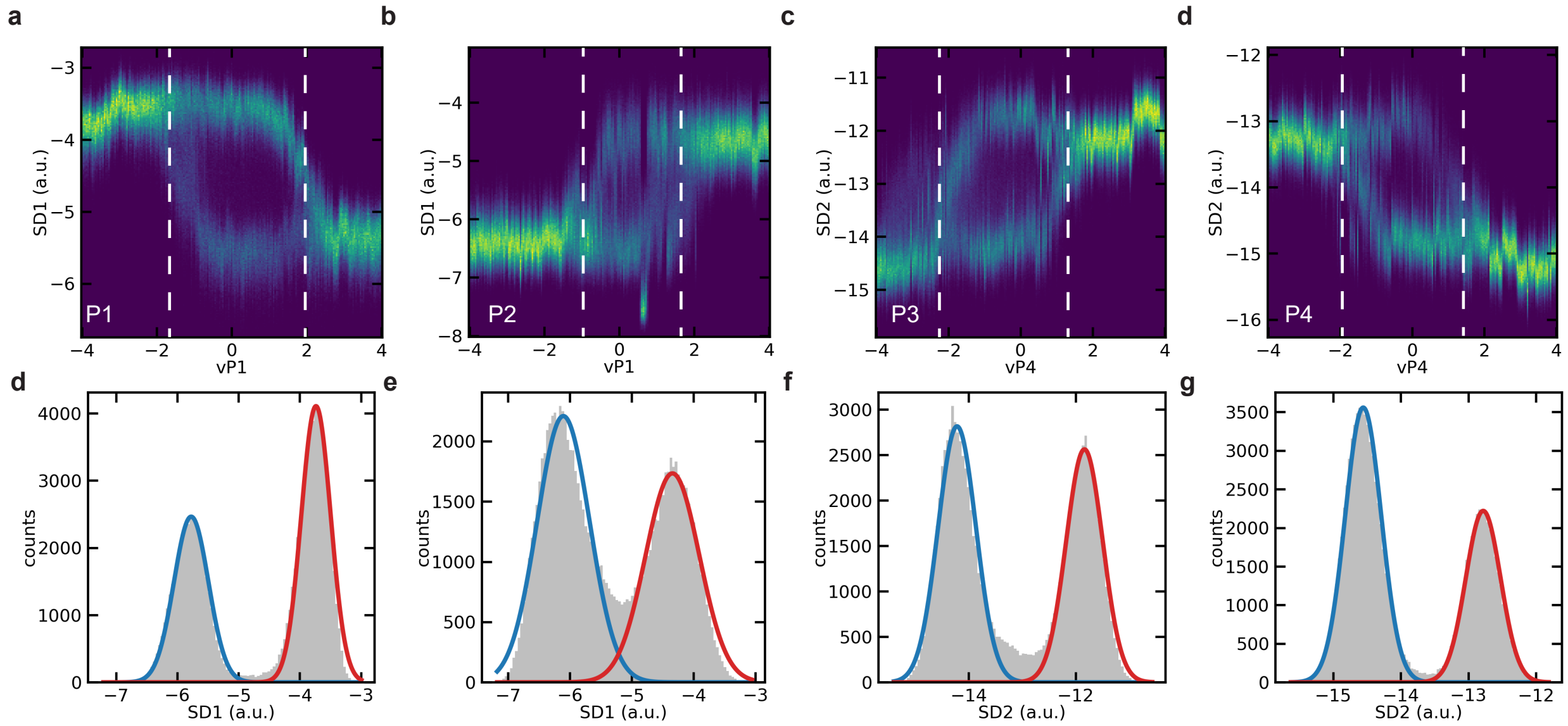

Figure S10: **Extended - PSB spectroscopy.** (a-d) Pauli Spin Blockade (PSB) spectroscopy for the four quantum dots (P1-P4). The white lines highlight the regions where the Singlet and Triplet states are associated with two distinguishable charge states and identify the so-called PSB window. (e-h) Histograms of the sensing dot signal at the center of the PSB window obtained from 100.000 single shots.

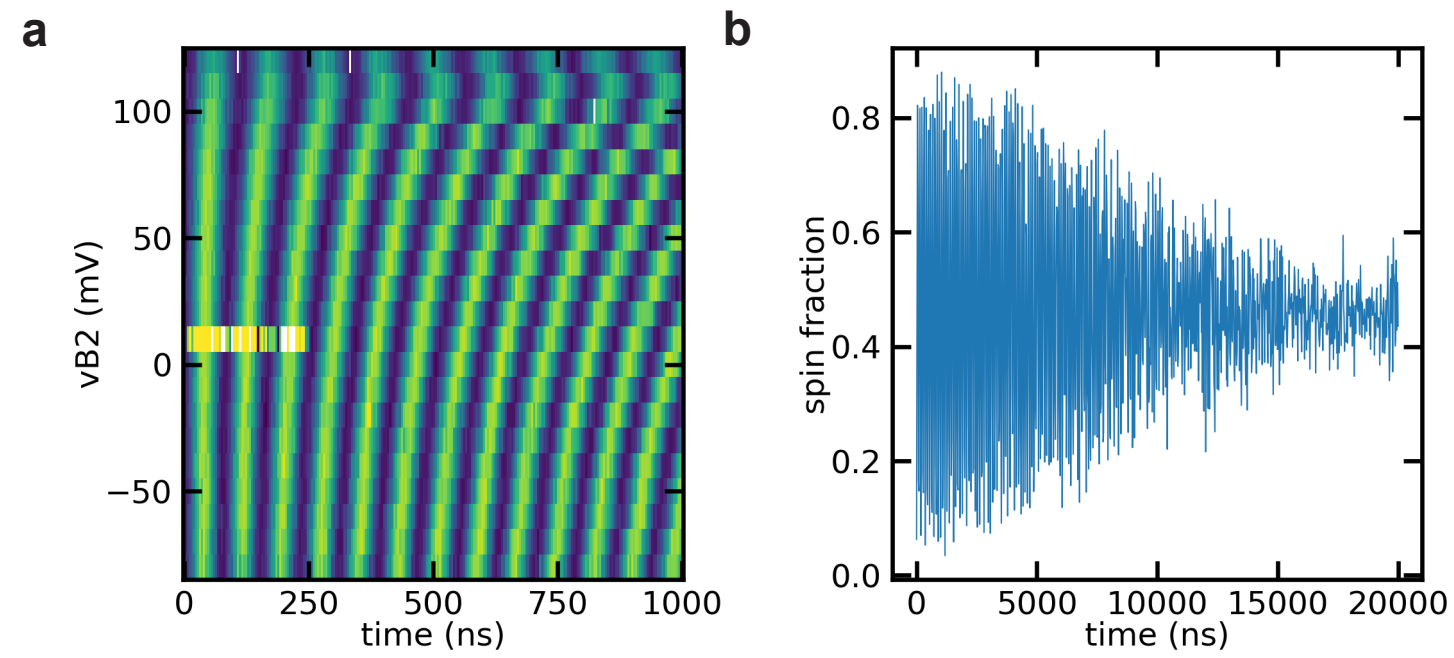

Figure S11: **Extended - Coherent oscillations.** Coherent oscillation of the spin fraction for the double quantum dot definer under P1 and P2 as a function of the barrier between the two dots (vB2) and of the free evolution time.

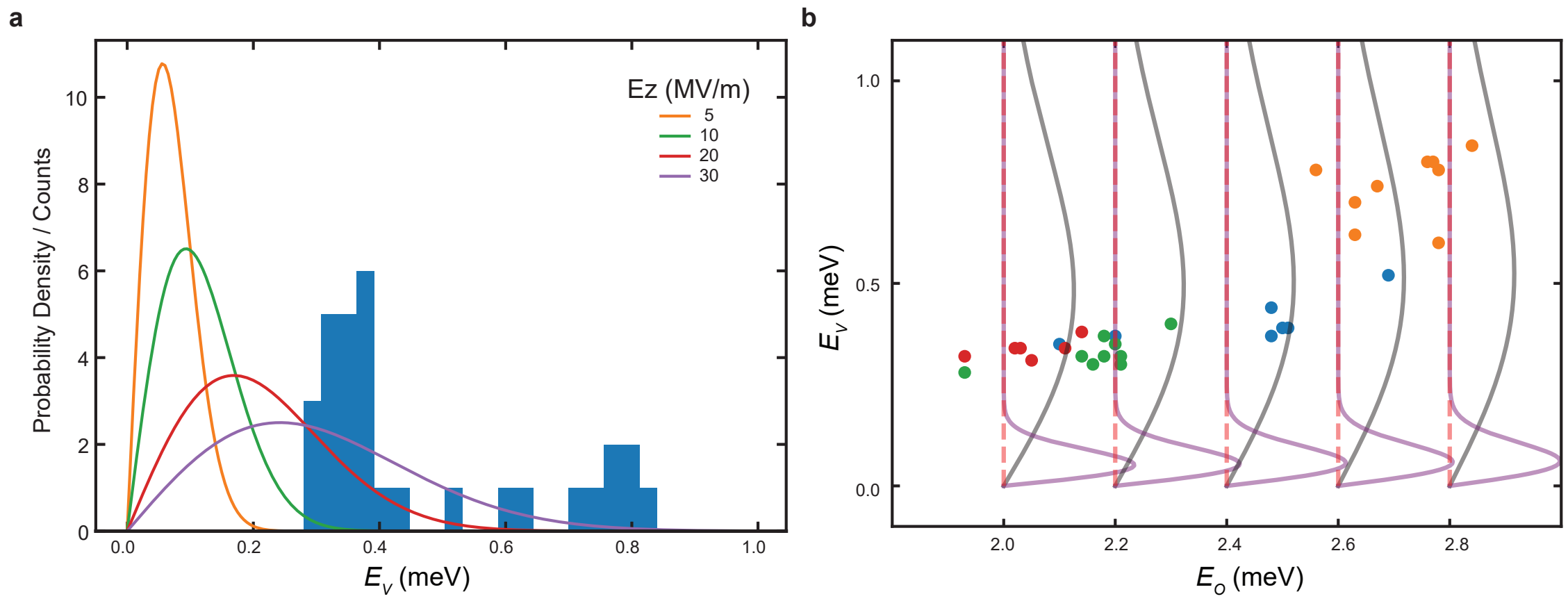

Figure S12: **Simulations of the Valley splitting distributions.** (a) Histogram of the measured single-electron valley splitting (E_{VS}) with associated Rayleigh distributions calculated assuming the measured average orbital energy of $\simeq 2.3$ meV and for different electric fields (E). From the characterization of the two-dimensional electron gas, we estimate a maximum electric field at the quantum well before tunneling to the oxide of $\simeq 5$ MV/m. (b) Cross-correlation plot between the orbital energy (E_O) and the single-electron valley splitting (E_V) for the four quantum dots (solid dots). The solid lines correspond to the associated Rayleigh distributions at a fixed orbital energy, and using the measured Ge concentration profile (purple) or leaving the y-z integral as a fitting parameter (gray). The z-y integral quantifies the alloy disorder experienced by electrons in the quantum well, which is predicted to be the dominant effect driving the valley splitting. In all simulations, we assume that the electron in the quantum well experiences a fixed electric field of $\simeq 5$ MV/m.

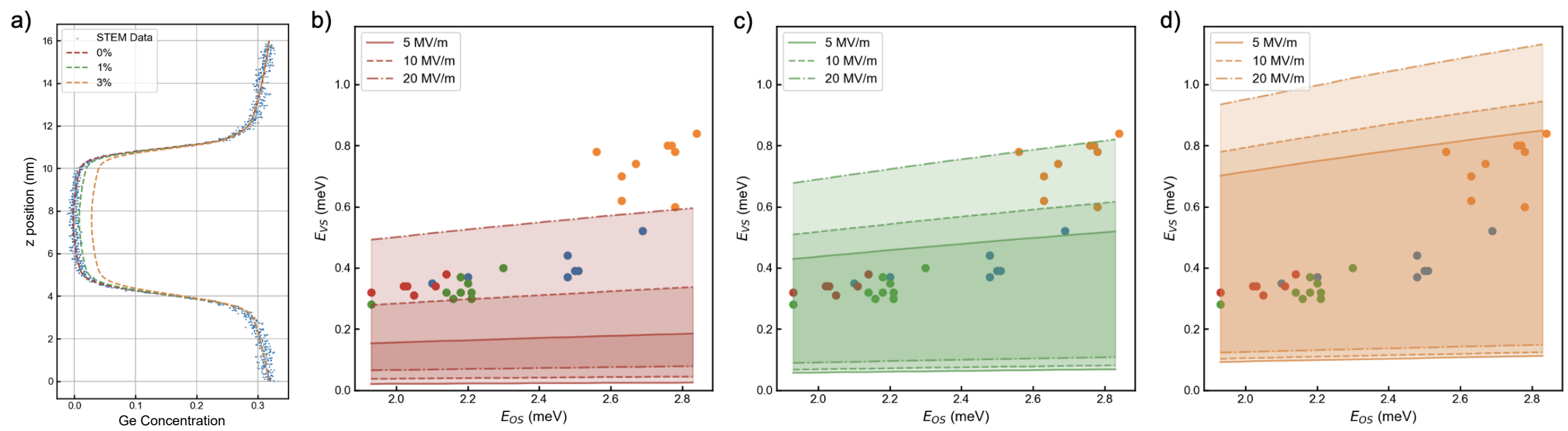

Figure S13: **Valley splitting distributions in the presence of a small Ge concentration in the quantum well.** (a) Ge concentration profile extracted by rescaling atomically-resolved STEM images of the ^{28}Si/SiGe heterostructure similar to Fig.S2. The chemical concentrations in the SiGe and quantum well are extracted from secondary ion mass spectroscopy (SIMS) and electron energy loss spectroscopy (EELS). We fit the experimental with a modified Sigmoid function (red) with 0% Ge concentration in the quantum well. Consequently, adjusted profiles (green and orange) are generated to mimic the presence of small Ge concentration (1% and 3%) in the quantum well. (b-d) Orbital projected $5^{th} - 95^{th}$ percentile valley splitting range for each concentration profile and different vertical electric fields (E_Z). At high electric field and even very small Ge concentration, most of the experimental data falls within the predicted valley splitting distribution.

Table S1: **Energy scales.** Summary of measured energy scales and lever arms for each dot in the four-dot device characterized in the main text.

Dot #	α (eV/V)	E_C (meV)	E_O (meV)	E_{V-1e} (meV)	E_{ST} (meV)	E_{PSB} (meV)
1	0.115(5)	7.02(5)	2.52(3)	0.55(1)	0.280(5)	0.42
2	0.103(4)	5.98(7)	2.77(4)	0.76(2)	0.315(7)	0.32
3	0.093(7)	5.16(8)	1.97(4)	0.39(1)	0.304(8)	0.33
4	0.090(8)	5.40(7)	2.02(5)	0.34(1)	0.202(10)	0.32

Table S2: **Pearson linear regression test.** Results of a Pearson linear regression test between single-electron valley splitting and orbital energy for the four dots in the quantum dot array. The Pearson correlation coefficient is normalized between −1 and 1 where 1 indicates perfect correlation −1 perfect anti-correlation, and 0 the absence of any correlation. For each test, we report the corresponding p-value to assess the statistical significance.

Dot #	Pearson correlation	p-value
1	0.76	0.05
2	0.31	0.42
3	0.69	0.04
4	0.67	0.14

References

(1) Sabbagh, D. et al. Quantum Transport Properties of Industrial $^{28}\mathrm{Si}/^{28}{\mathrm{Si}\mathrm{O}}_{2}$. *Physical Review Applied* **2019**, *12*, 014013.

(2) Degli Esposti, D.; Stehouwer, L. E. A.; Gül, Ö.; Samkharadze, N.; Déprez, C.; Meyer, M.; Meijer, I. N.; Tryputen, L.; Karwal, S.; Botifoll, M.; others Low disorder and high valley splitting in silicon. *npj Quantum Information* **2024**, *10*, 32.

(3) Degli Esposti, D.; Paquelet Wuetz, B.; Fezzi, V.; Lodari, M.; Sammak, A.; Scappucci, G. Wafer-scale low-disorder 2DEG in ^{28}Si/SiGe without an epitaxial Si cap. *Applied Physics Letters* **2022**, *120*, 184003.

(4) Paquelet Wuetz, B. et al. Atomic fluctuations lifting the energy degeneracy in Si/SiGe quantum dots. *Nature Communications* **2022**, *13*, 7730.

(5) Undseth, B.; Meggiato, N.; Wu, Y.-H.; Katiraee-Far, S. R.; Tryputen, L.; de Snoo, S. L.; Degli Esposti, D.; Scappucci, G.; Greplová, E.; Vandersypen, L. M. K. Weight-four parity checks in a spin-shuttling architecture. *Nature* **2026**, *655*, 1160–1166.

(6) De Smet, M.; Matsumoto, Y.; Zwerver, A.-M. J.; Tryputen, L.; de Snoo, S. L.; Amitonov, S. V.; Katiraee-Far, S. R.; Sammak, A.; Samkharadze, N.; Gül, Ö.; others High-fidelity single-spin shuttling in silicon. *Nature Nanotechnology* **2025**, *20*, 866–872.

(7) Matsumoto, Y.; De Smet, M.; Tryputen, L.; de Snoo, S. L.; Amitonov, S. V.; Sammak, A.; Rimbach-Russ, M.; Scappucci, G.; Vandersypen, L. M. K. Two-qubit logic and teleportation with mobile spin qubits in silicon. *Nature* **2026**, *653*, 391–397.

(8) Undseth, B. et al. Hotter is easier: Unexpected temperature dependence of spin qubit frequencies. *Physical Review X* **2023**, *13*, 041015.

(9) Philips, S. G. J.; Mądzik, M. T.; Amitonov, S. V.; de Snoo, S. L.; Rimbach-Russ, M.; Kalhor, N.; Volk, C.; Lawrie, W. I. L.; Brousse, D.; Tryputen, L.; Paquelet Wuetz, B.; Sammak, A.; Veldhorst, M.; Scappucci, G.; Vandersypen, L. M. K. Universal control of a six-qubit quantum processor in silicon. *Nature* **2022**, *609*, 919–924.

(10) Dodson, J. P.; Ercan, H. E.; Corrigan, J.; Losert, M. P.; Holman, N.; McJunkin, T.; Edge, L. F.; Friesen, M.; Coppersmith, S. N.; Eriksson, M. A. How valley-orbit states in silicon quantum dots probe quantum well interfaces. *Physical Review Letters* **2022**, *128*, 146802.

(11) Losert, M. P.; Eriksson, M. A.; Joynt, R.; Rahman, R.; Scappucci, G.; Coppersmith, S. N.; Friesen, M. Practical strategies for enhancing the valley splitting in Si/SiGe quantum wells. *Physical Review B* **2023**, *108*, 125405.

(12) Marcks, J. C.; Eagen, E.; Brann, E. C.; Losert, M. P.; Oh, T.; Reily, J.; Wang, C. S.; Keith, D.; Mohiyaddin, F. A.; Luthi, F.; others Valley splitting correlations across a silicon quantum well containing germanium. *Nature Communications* **2025**, *16*, 11381.
